# Changes in regulators of lipid metabolism in the brain: a study of animal models of depression and hypothyroidism

**DOI:** 10.1007/s43440-022-00395-8

**Published:** 2022-08-11

**Authors:** Katarzyna Głombik, Jan Detka, Magdalena Kukla-Bartoszek, Alicja Maciejska, Bogusława Budziszewska

**Affiliations:** 1 Laboratory of Immunoendocrinology, Department of Experimental Neuroendocrinology, Maj Institute of Pharmacology, Polish Academy of Sciences, Smętna 12, 31-343 Kraków, Poland; 2 Department of Biochemical Toxicology, Chair of Toxicology, Medical College, Jagiellonian University, Medyczna 9, 30-688 Kraków, Poland

**Keywords:** Brain lipid biosynthesis, Cholesterol, Fatty acids, Depression, Hypothyroidism

## Abstract

Metabolic disturbances in the brain are assumed to be early changes involved in the pathogenesis of depression, and these alterations may be intensified by a deficiency of thyroid hormones. In contrast to glucose metabolism, the link between altered brain lipids and the pathogenesis of depression is poorly understood, therefore in the present study, we determine transcription factors and enzymes regulating cholesterol and fatty acid biosynthesis in the brain structures in an animal model of depression, hypothyroidism and the coexistence of these diseases.

In used model of depression, a decrease in the active form of the transcription factor SREBP-2 in the hippocampus was demonstrated, thus suggesting a reduction in cholesterol biosynthesis. In turn, in the hypothyroidism model, the reduction of cholesterol biosynthesis in the frontal cortex was demonstrated by both the reduction of mature SREBP-2 and the concentration of enzymes involved in cholesterol biosynthesis. The lower expression of LDL receptors in the frontal cortex indicates the restriction of cholesterol uptake into the cells in the model of coexistence of depression and hypothyroidism. Moreover, the identified changes in the levels of SNAP-25, GLP-1R and GLP-2R pointed to disturbances in synaptic plasticity and neuroprotection mechanisms in the examined brain structures.

In conclusion, a reduction in cholesterol synthesis in the hippocampus in the model of depression may be the reason for the reduction of synaptic plasticity, whereas a lower level of LDL-R occurring in the frontal cortex in rats from the model of depression and hypothyroidism coexistence could be the reason of anxiogenic and depression-like behaviors.

**Supplementary Information:**

The online version contains supplementary material available at 10.1007/s43440-022-00395-8.

## Introduction

Despite many studies, the pathogenesis of depression is still poorly understood and, consequently, the treatment of this disease is ineffective in many patients. More and more data indicate that currently used antidepressants, which mainly affect the neurotransmission disturbed in this disease, are not fully effective because they do not normalize the processes leading to changes in neurotransmission, such as metabolic, hormonal, and immunological disruptions in the brain [1–4]. In particular, many data indicate that metabolic disturbances in the brain may be the cause of an insufficient energy supply for the proper synthesis, release, and function of neurotransmitters [5, 6]. Moreover, it is believed that the determination of the metabolic changes in the brain in depressive disorders could provide a new, more effective treatment target. In fact, until now studies using functional neuroimaging have shown altered brain glucose metabolism in depressed patients and in animal models of depression, plus changes in the amount or the activity of key metabolic enzymes, factors regulating glucose metabolism, and insulin resistance in certain regions of the brain have been identified [1, 7]. In the context of metabolic disturbances in depression, a decreased level or function of thyroid hormones in the brain may play an important role. The role of thyroid hormones in the pathogenesis of depression is evidenced by epidemiological data showing the frequent coexistence of depression and hypothyroidism, similar clinical effects observed in these diseases, including cognitive dysfunction, and the effectiveness of thyroid hormone adjunctive therapy in the drug-resistant one and bipolar depression [8–11]. Although most of the genes regulated by thyroid hormones in the developing brain are insensitive to them in the adult brain, it is now known that thyroid hormones are also needed for a properly functioning central nervous system (CNS) in adults, where they regulate genes encoding proteins involved in metabolic processes, synaptic plasticity, and learning and memory processes [12]. Adult-onset hypothyroidism also impairs the cholinergic system, presynaptic proteins involved in neurotransmitters’ release, phosphatases activity, and adult hippocampal neurogenesis [12, 13]. Our previous studies have shown, inter alia, that in the animal model of the coexistence of depression and hypothyroidism there is a stronger weakening of the mechanism of presynaptic neurotransmitter release than in models of depression or hypothyroidism alone [14].

In contrast to the changes in glucose metabolism in depression and the effects of hypothyroidism on the carbohydrate metabolism and synaptic plasticity in this disease, the potential role of disturbances in brain lipid metabolism in the pathogenesis of depression is very poorly understood, and in models of the coexistence of depression and hypothyroidism this has not been the subject of research so far. Lipids, as key components of cell membranes, regulate a lot of neuronal functions, including membrane fluidity and permeability, vesicle formation and transport, neurotransmitter release, cell integrity and plasticity. Some data indicate that disturbance of lipid homeostasis may play an important role in the pathogenesis of both neurodegenerative diseases and mood disorders [15].

Brain lipids mainly come from de novo biosynthesis as the blood–brain barrier prevents them from passing from the periphery to the brain. The biosynthesis of fatty acids and cholesterol is controlled by the sterol regulatory element binding proteins’ (SREBP-1 and SREBP-2) transcription factors. Although the functions of different SREBP isoforms overlap, SREBP-1 preferentially regulates fatty acid biosynthesis, and SREBP-2 mainly regulates cholesterol synthesis [16]. These transcription factors exist as inactive precursors (120 kDa) in a complex with the SREBP cleavage activating protein (SCAP) and the insulin induced gene (INSIG-1 and INSIG-2) proteins. Upon activation by proteolysis, SREBP (60–70 kDa) are translocated to the nucleus, bind to the sterol regulatory element (SRE), and activate lipogenic genes. Disturbances in brain lipid homeostasis have so far been demonstrated in neurodegenerative diseases, including Alzheimer’s and Parkinson’s as well as in schizophrenia [17, 18]. In the case of depression, the results of preclinical studies also suggest the role of fatty acids in the pathogenesis of this disease, and moreover, it has been shown that some antidepressants, such as antipsychotics, increase the synthesis of cholesterol and fatty acids [15, 19]. Furthermore, current studies also show that in depression, disturbances in glial cells, including astrocytes, which are the main site of cholesterol and fatty acid synthesis, occur earlier and that their dysfunction leads to changes in synaptic plasticity, possibly resulting in the neurotransmission alteration observed in depression [20]. Since lipid-containing neuronal membranes in the brain are not fixed structures, their lipid composition may have a significant influence on neuronal function and signaling. Cholesterol is involved in the synaptogenesis process, which is disturbed in depression, and is also an important component of myelin. Because the reduced density of myelin-producing oligodendrocytes in the prefrontal cortex of patients with major depression has been shown, the determination of their biosynthesis alongside fatty acids in the depression model with and without hypothyroidism may contribute to a better understanding of the pathogenesis of this disease and, consequently, also improve its therapy [21].

The aim of the present research was to determine the factors regulating the biosynthesis of cholesterol and fatty acids in the model of depression and hypothyroidism, and the model of the coexistence of these diseases. Wistar-Kyoto (WKY) rats, which exhibit hormonal and behavioral characteristics similar to those found in depression, including increased stress reactivity and dysregulation of the hypothalamic–pituitary–adrenal axis (HPA), were used as a model of depression [22]. Moreover, this strain of rats is considered to be a model of depression resistant to antidepressant therapy [23]. The hypothyroidism model was induced by administration of a thyroid peroxidase inhibitor—propylthiouracil (PTU). Selected markers of lipid biosynthesis and uptake were compared between WKY rats and control Wistar rats under basal conditions and under conditions of hypothyroidism. The frontal cortex and the hippocampus were selected for this study as they are the main structures affected by depressive disorders.

## Materials and methods

### Animals and treatments

All procedures involving animals were approved by the Local Ethics Committee, Kraków, Poland (Permit No. 46/2018 of 01.02.2018).

Male Wistar and Wistar-Kyoto rats (aged 8 weeks upon arrival) were obtained from Charles-River Laboratories (Charles River Laboratories, Hamburg, Germany) and then kept in a natural day/night cycle at room temperature (23 °C) with free access to food and water. After an acclimatization period, the rats were randomly assigned into the following four groups: Group I (Control): Wistar rats drinking water ad libitum; Group II (Endogenous depression): Wistar-Kyoto rats drinking water ad libitum; Group III (Hypothyroidism): Wistar rats treated with 0.05% (w/v) PTU in drinking water for 3 weeks; and Group IV (Coexistent depression and hypothyroidism): Wistar-Kyoto rats treated with 0.05% (w/v) PTU in drinking water for 3 weeks.

### Tissue processing procedure

After 3 weeks of treatment, the rats were sacrificed between 9:00 a.m. and 1:00 p.m. under non-stressful conditions by rapid decapitation to isolate biological material (the frontal cortex and hippocampus) for further research. The dissection of the frontal cortex was carried out based on a study by Chiu et al. [24]. The whole hippocampus was taken for analysis.

The brain structures were dissected on ice-cold glass plates, frozen on dry ice and stored at −80 °C.

### Western blot analysis of tissue homogenates

Samples were isolated and processed according to a protocol described previously [14]. In brief, brain structures such as the frontal cortex and hippocampus were homogenized in 2% SDS (BioShop, Burlington, ON, Canada) and prepared for the following analysis by mixing equal amounts of protein with loading buffer (Bio-Rad, CA, Hercules, CA, USA) before being boiled (5 min, 95 °C). Then, the separation of proteins (~1 h, constant voltage of 150 V) and semi-dry transfer to PVDF membranes (Sigma-Aldrich, Saint Louis, MO, USA) were conducted. After that, the membranes were blocked with 5% skimmed milk in Tris-buffered saline (TBS) with 0.05% Tween 20 (Sigma-Aldrich, Saint Louis, MO, USA); (1 h, room temperature) and incubated with the primary antibodies (overnight, 4 °C). The following antibodies were used: anti-GFAP (Abcam, Cambridge, UK, 1:10,000), anti-SREBP-1 (ThermoFisher Scientific, Waltham, MA, USA, 1 µg/ml), anti-SREBP-2 (ThermoFisher Scientific, Waltham, MA, USA; 1:1000), anti-INSIG-1 (Novus Biologicals, Littleton, CO, USA; 1:1000), anti-INSIG-2 (Novus Biologicals, Littleton, CO, USA, 1:250), anti-LDL-R (Bioassay Technology Laboratory, Shanghai, China, 1:1000). Some membranes were cut to allow simultaneous incubation with different antibodies. The next day, the membranes were washed in TBS with 0.1% Tween 20 (TBS-T; 4 times, 10 min each), then incubated with HRP peroxidase-conjugated appropriate secondary antibody: horse anti-mouse and goat anti-rabbit IgG HRP peroxidase-conjugated secondary antibody (Vector Laboratories, Peterborough, UK, 1:4000); (1 h, room temperature) and subjected to the second round of washing in TBS-T (4 times, 10 min each). The bands were detected using BM Chemiluminescence Western Blotting Substrate (POD) (Roche, Mannheim, Germany) and visualized using a luminescent image analyzer with a Fujifilm LAS-1000 System (Fujifilm, Tokyo, Japan). Densitometric measurements were applied for the quantification of the relative levels of protein concentration using Fujifilm Multi Gauge software (Fujifilm, Tokyo, Japan). Antibody anti-β-actin (Sigma-Aldrich, Saint Louis, MO, USA, 1:12,000) or Vinculin (Sigma-Aldrich, Saint Louis, MO, USA, 1:15,000) were used as an internal loading control. Some membranes were stripped and reprobed.

### ELISA analysis of tissue homogenates

The levels of 3-Hydroxy-3-Methylglutaryl Coenzyme A Reductase—HMGCR, 3-Hydroxy-3-Methylglutaryl Coenzyme A Synthase—HMGCS, Stearoyl Coenzyme A Desaturase—SCD, Synaptosomal Associated Protein 25 kDa—SNAP-25, Necdin (all from ELK (Wuhan) Biotechnology CO., Ltd., Wuhan, China) and Glucagon-like Peptide Receptors—GLP-1R and GLP-2R (FineTest, Wuhan, China) in the frontal cortices and hippocampal homogenates were analyzed using commercially available ELISA kits according to the instructions provided by the manufacturers. For each ELISA, the samples were prepared in accordance with the supplier’s recommendations. The absorbance was measured using a Tecan Infinite M200 Pro (TECAN, Männedorf, Switzerland) set to the appropriate wavelength.

## Statistics

All graphs were prepared using GraphPad Prism 8. Statistical analysis was performed using Statistica 13.3 software and consisted of two-way analyses of variance (ANOVAs) followed by the Duncan post-hoc test when appropriate. Differences were considered significant at *p* < 0.05. The ANOVA results are reported as an F-statistic and its associated degrees of freedom.

## Results

### Level of glial fibrillary acidic protein (GFAP)

**Fig. 1:**
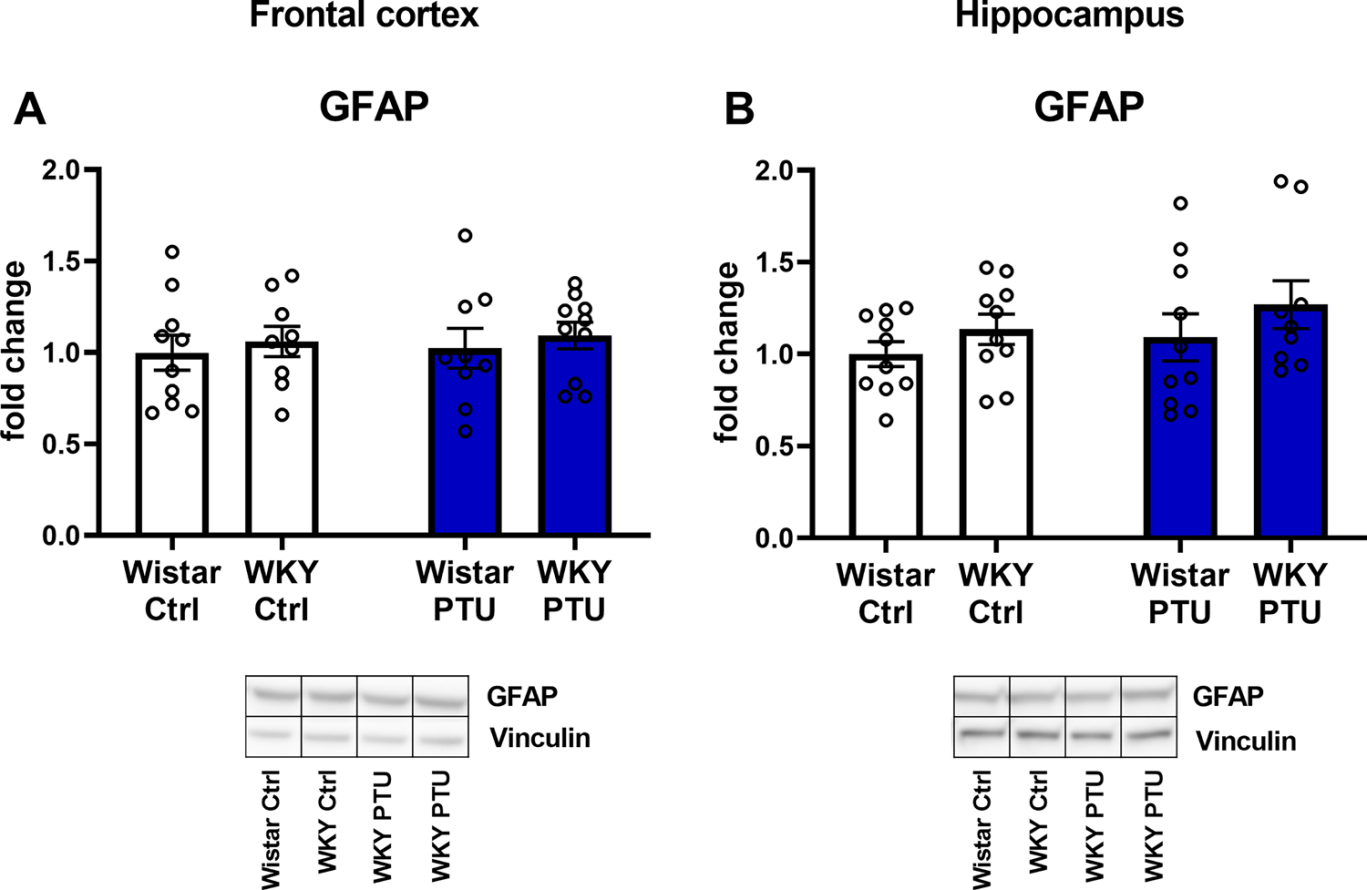
The effects of strain and PTU treatment on the protein level of GFAP in the frontal cortex **A** and hippocampus **B**. The results are expressed as the average fold change ± SEM. *n* = 9–10. Statistics: two-way ANOVA

No changes in the protein level of GFAP were detected either in the frontal cortex or in the hippocampus (Fig. 1).

### Levels of sterol regulatory element-binding protein 1 and 2 (SREBP-1 and SREBP-2)

**Fig. 2:**
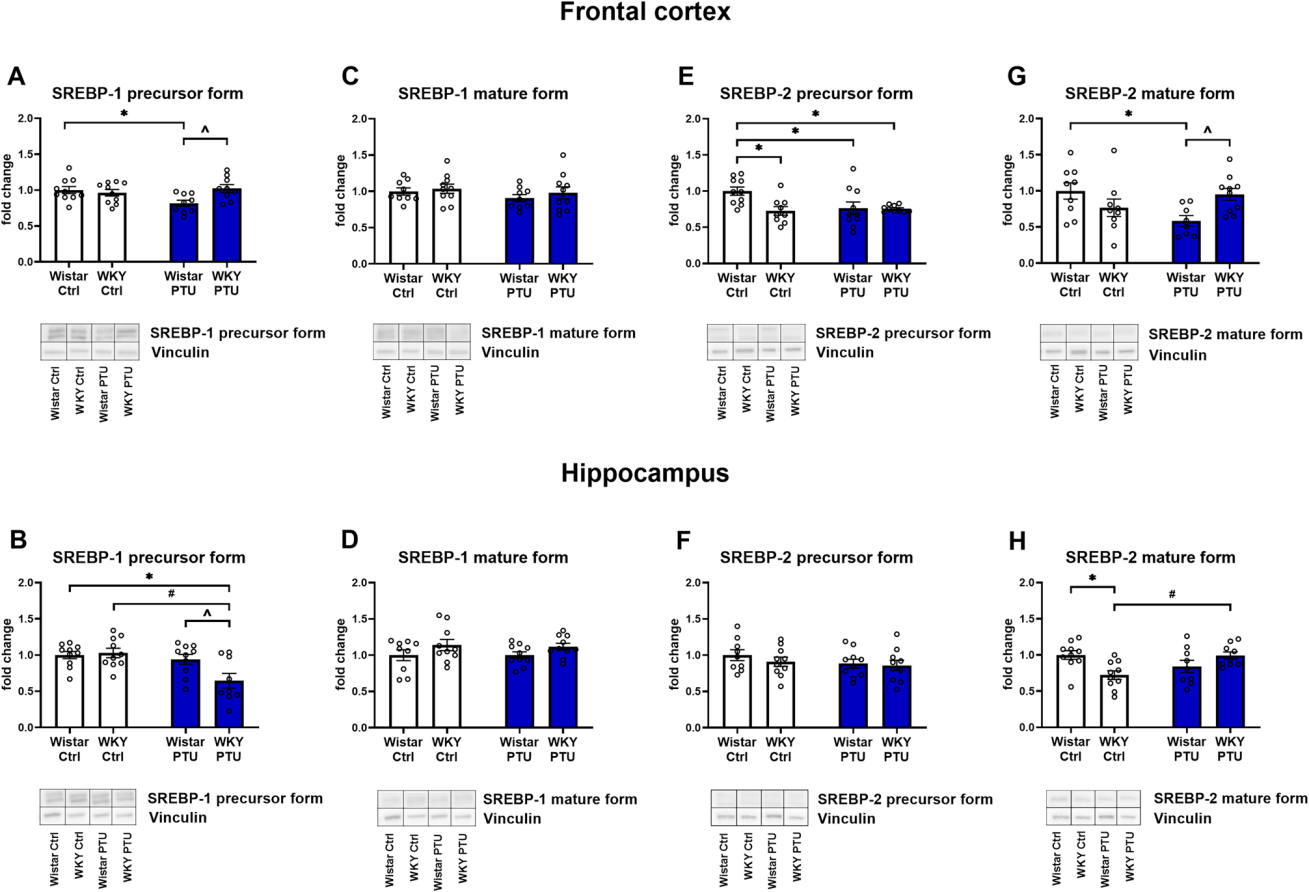
The effects of strain and PTU treatment on the protein levels of precursor form **A**, **B** and mature form **C**, **D** of SREBP-1 and precursor **E**, **F** and mature form **G**, **H** of SREBP-2 in the frontal cortex and hippocampus. The results are expressed as the average fold change ± SEM. *p < 0.05 vs. the control group (Wistar rats); #*p* < 0.05 vs. the WKY group, ^ vs. the Wistar + PTU group. *n* = 8–10. Statistics: two-way ANOVA followed by Duncan’s test

PTU administration led to a reduction in SREBP-1 precursor form in the frontal cortex of Wistar rats (strain × PTU interaction: *F*_1,34_ = 6.34, *p* = 0.017, whereas no changes between the groups were detected in the case of the mature form (Fig. 2A, C). SREBP-1 precursor in the hippocampus was downregulated by PTU only in WKY rats (PTU effect *F*_1,35_ = 9.61, *p* < 0.05; strain × PTU interaction: *F*_1,35_ = 5.30, *p* = 0.027) in comparison to all of the other groups (Fig. 2B). As in the case of the frontal cortex, there was no difference between the groups in mature SREBP-1 (Fig. 2D) in the hippocampus.

The precursor of SREBP-2 in the frontal cortex was diminished in all of the examined groups (WKY animals, Wistar with PTU and WKY with PTU) in comparison to control (strain × PTU interaction: *F*_1,35_ = 4.87, *p* = 0.034) (Fig. 2E). Furthermore, analysis also revealed the strain × PTU interaction (*F*_1,32_ = 9.13, *p* = 0.005) after having taken into account the mature SREBP-2 in this brain area. The Wistar PTU group was characterized by the lower level of this protein when compared to non-treated rats (Fig. 2G).

No changes in the precursor form of SREBP-2 were detected in the hippocampus in either of the examined groups, whereas the interaction of the strain × PTU was observed in data analysis of mature SREBP-2 (*F*_1,34_ = 11.16, *p* = 0.002) (Fig. 2F, H). SREBP-2 was lowered in WKY rats without PTU administration. Reductions in precursor form of SREBP-1 and mature form of SREBP-2 levels in the frontal cortex in PTU-treated Wistar rats and in the concentration of mature SREBP-2 form in the hippocampus of WKY animals were attenuated in a model of comorbid depression and hypothyroidism.

### Levels of insulin-induced gene 1 protein (INSIG-1) and insulin-induced gene 2 protein (INSIG-2)

There were no changes in the levels of INSIG-1 and INSIG-2 between the groups in both of the studied brain areas (Table 1).

**Table 1:** The effects of strain and PTU treatment on the protein level of INSIG-1 and INSIG-2 in the frontal cortex and hippocampus

Protein	Brain structure	Wistar	Wistar-Kyoto	Wistar PTU	Wistar -Kyoto PTU
INSIG-1	Frontal cortex	1.00 ± 0.04	0.94 ± 0.08	0.92 ± 0.08	0.87 ± 0.09
	Hippocampus	1.00 ± 0.07	1.08 ± 0.11	0.96 ± 0.15	0.94 ± 0.07
INSIG-2	Frontal cortex	1.00 ± 0.09	0.91 ± 0.07	0.93 ± 0.09	0.97 ± 0.07
	Hippocampus	1.00 ± 0.10	1.15 ± 0.10	1.09 ± 0.08	1.03 ± 0.08

The results are expressed as the average fold change ± SEM. *n* = 9–10. Statistics: two-way ANOVA

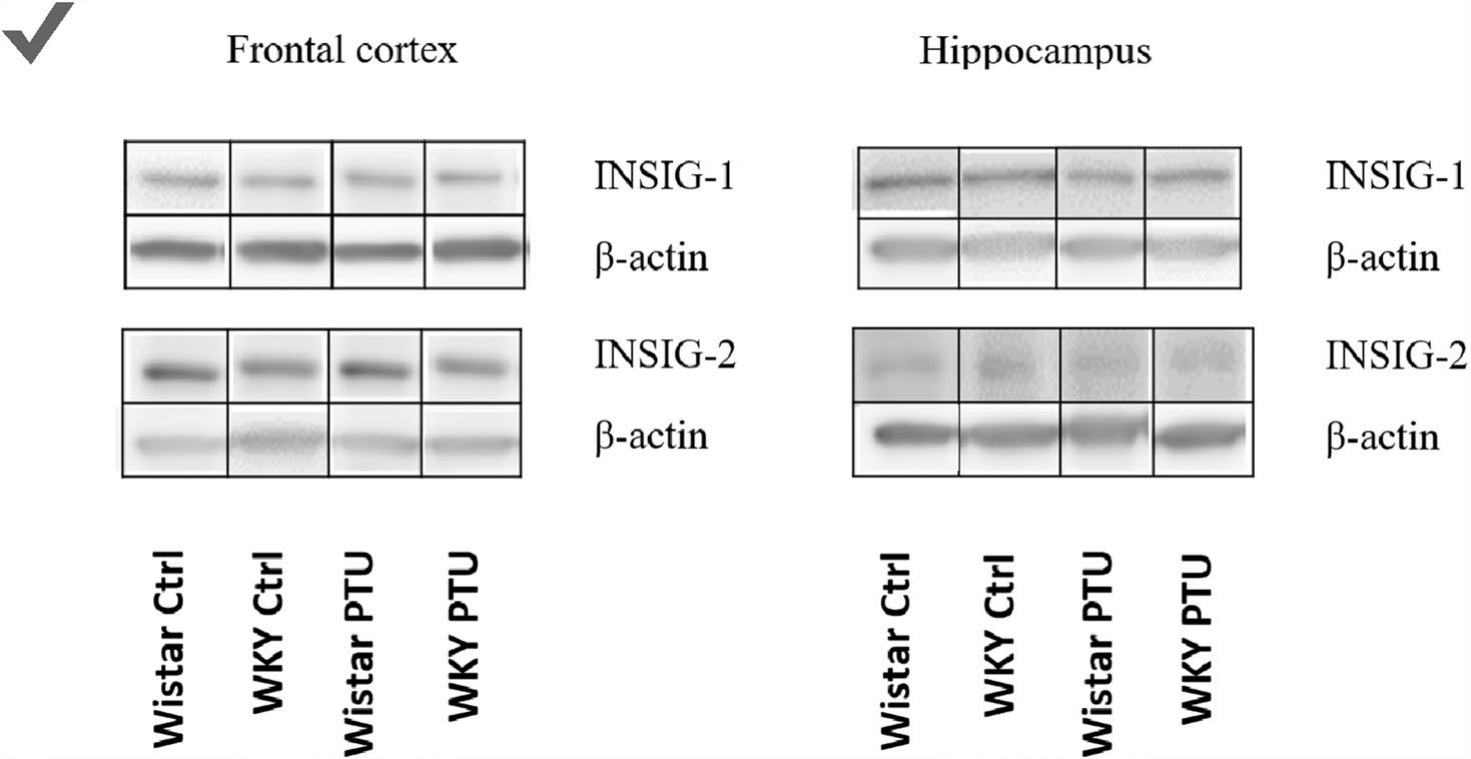

### Levels of 3-hydroxy-3-methyl-glutaryl-coenzyme A reductase (HMGCR) and 3-hydroxy-3-methyl-glutaryl- coenzyme A synthase (HMGCS)

**Fig. 3:**
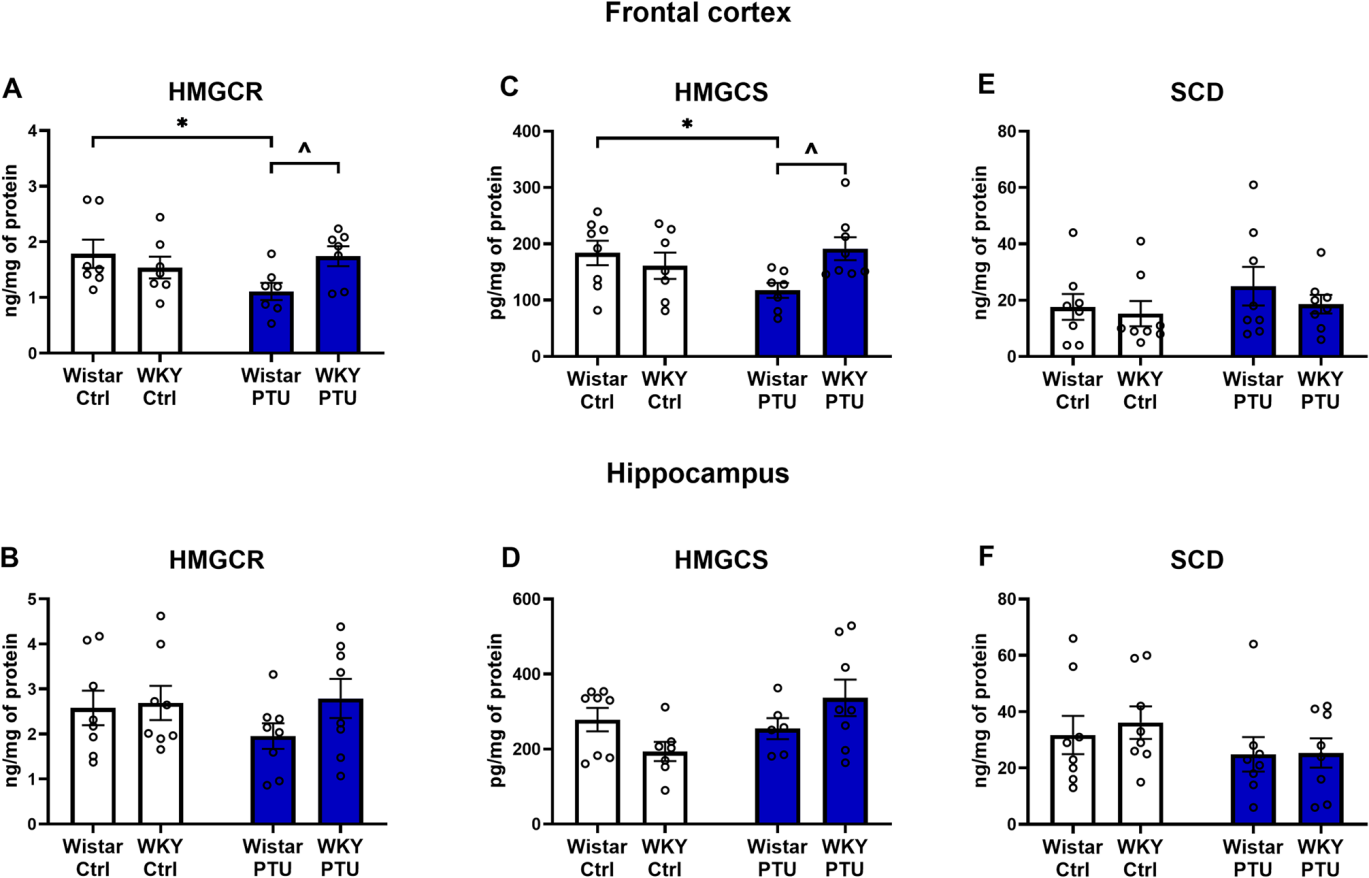
The effects of strain and PTU treatment on the protein levels of HMGCR **A**, **B**, HMGCS **C**, **D** and SCD **E**, **F** in the frontal cortex and hippocampus. The results are expressed as the pg/mg of protein or ng/mg of protein ± SEM. *p < 0.05 vs. the control group (Wistar rats), ^ vs. the Wistar PTU group. *n* = 6–8. Statistics: two-way ANOVA followed by Duncan’s test

In the frontal cortex, the interaction of strain × PTU was observed in the case of both of the measured factors (*F*_1,23_ = 4.87 for HMGCR, *p* = 0.037 and *F*_1, 26_ = 5.75 for HMGCS, *p* = 0.024) (Fig. 3A, C). In this brain structure, PTU diminished the level of HMGCR and HMGCS vs. control rats and these decreases were inhibited in the depression-hypothyroidism comorbidity model. In the hippocampus, there were no changes in the protein levels of HMGCR and HMGCS (Fig. 3B, D).

### Level of stearoyl-CoA desaturase (SCD)

No changes in the protein level of SCD between the study groups were detected either in the frontal cortex or in the hippocampus (Fig. 3E, F).

### Levels of low-density lipoprotein receptor (LDL-R) precursor (120 kDa) and mature 160 kDa form

**Fig. 4:**
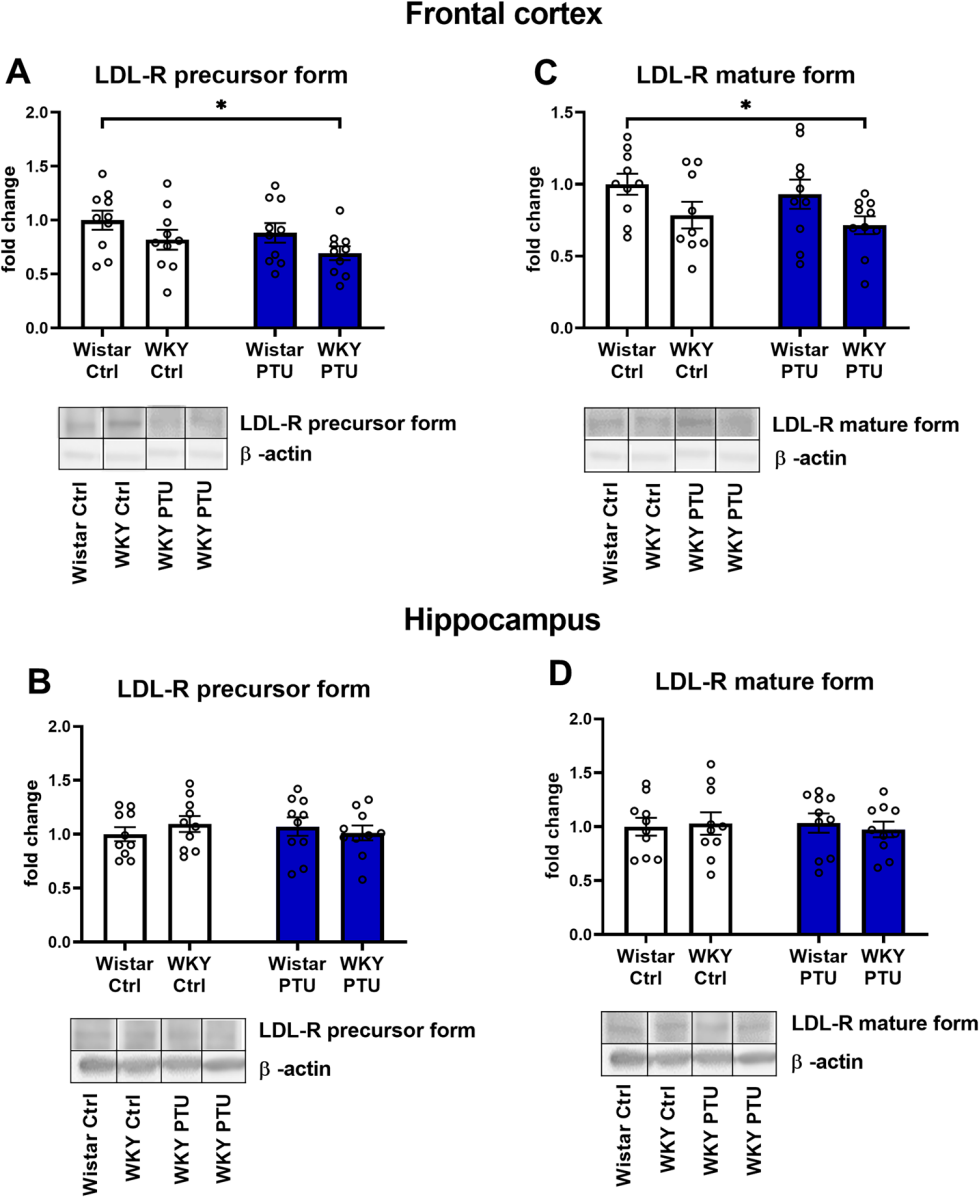
The effects of strain and PTU treatment on the protein level of LDL-R precursor form **A**, **B**, and mature form **C**, **D** in the frontal cortex and hippocampus. The results are expressed as the average fold change ± SEM. *p < 0.05 vs. the control group (Wistar rats). *n* = 8–10. Statistics: two-way ANOVA followed by Duncan’s test

Measuring the precursor form of LDL-R protein in the frontal cortex, the diminished level of this factor was observed in WKY rats treated with PTU (strain effect: *F*_1,36_ = 4.76, *p* = 0.036) (Fig. 4A). Moreover, a study on the mature form of this receptor also revealed that in WKY rats treated with PTU the investigated protein was decreased vs. Wistar animals (strain effect: *F*_1,35_ = 6.67, *p* = 0.014) (Fig. 4C). In the hippocampus a similar effect has not been demonstrated (Fig. 4B, D).

### Levels of presynaptic factors: Synaptosomal-associated protein 25 (SNAP-25) and Necdin

**Fig. 5:**
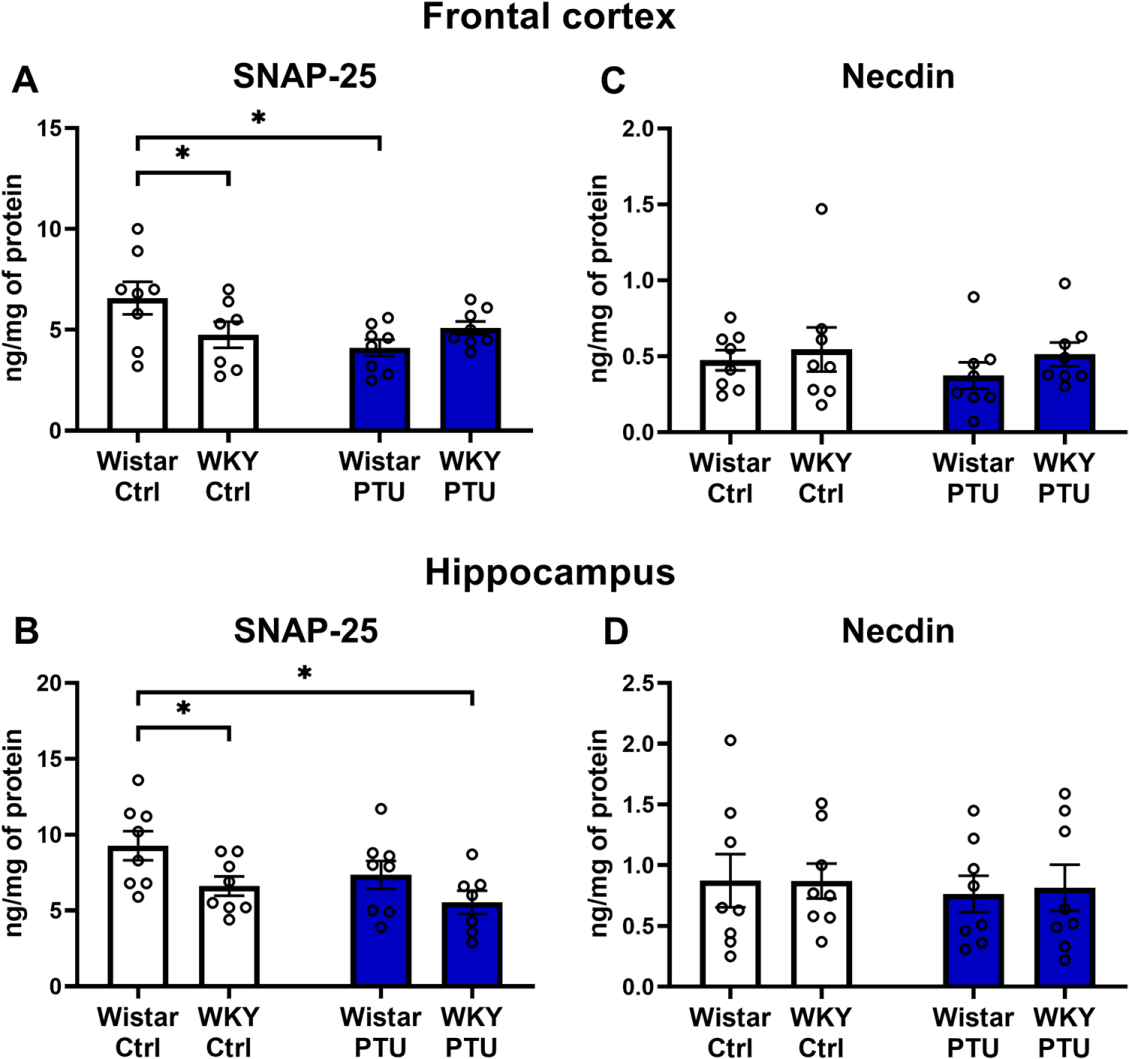
The effects of strain and PTU treatment on the protein levels of SNAP-25 **A**, **B** and Necdin **C**, **D** in the frontal cortex and hippocampus. The results are expressed as the ng/mg of protein ± SEM. *p < 0.05 vs. the control group (Wistar rats). *n* = 7–8. Statistics: two-way ANOVA followed by Duncan’s test

In the frontal cortex, SNAP-25 was reduced in WKY rats and Wistar animals treated with PTU when compared to control Wistar rats (strain × PTU interaction *F*_1,27_ = 15.84, *p* = 0.022) (Fig. 5A). In the hippocampus the strain effect was observed (*F*_1,27_ = 7.17, *p* = 0.012)—WKY rats (treated and non-treated with PTU) were characterized by a lower level of SNAP-25 than the control Wistar animals (Fig. 5B).

There were no changes in the protein levels of Necdin in either of the examined brain structures (Fig. 5C, D).

### Levels of glucagon-like peptide-1 receptor and glucagon-like peptide-2 receptor (GLP-1R and GLP-2R)

**Fig. 6:**
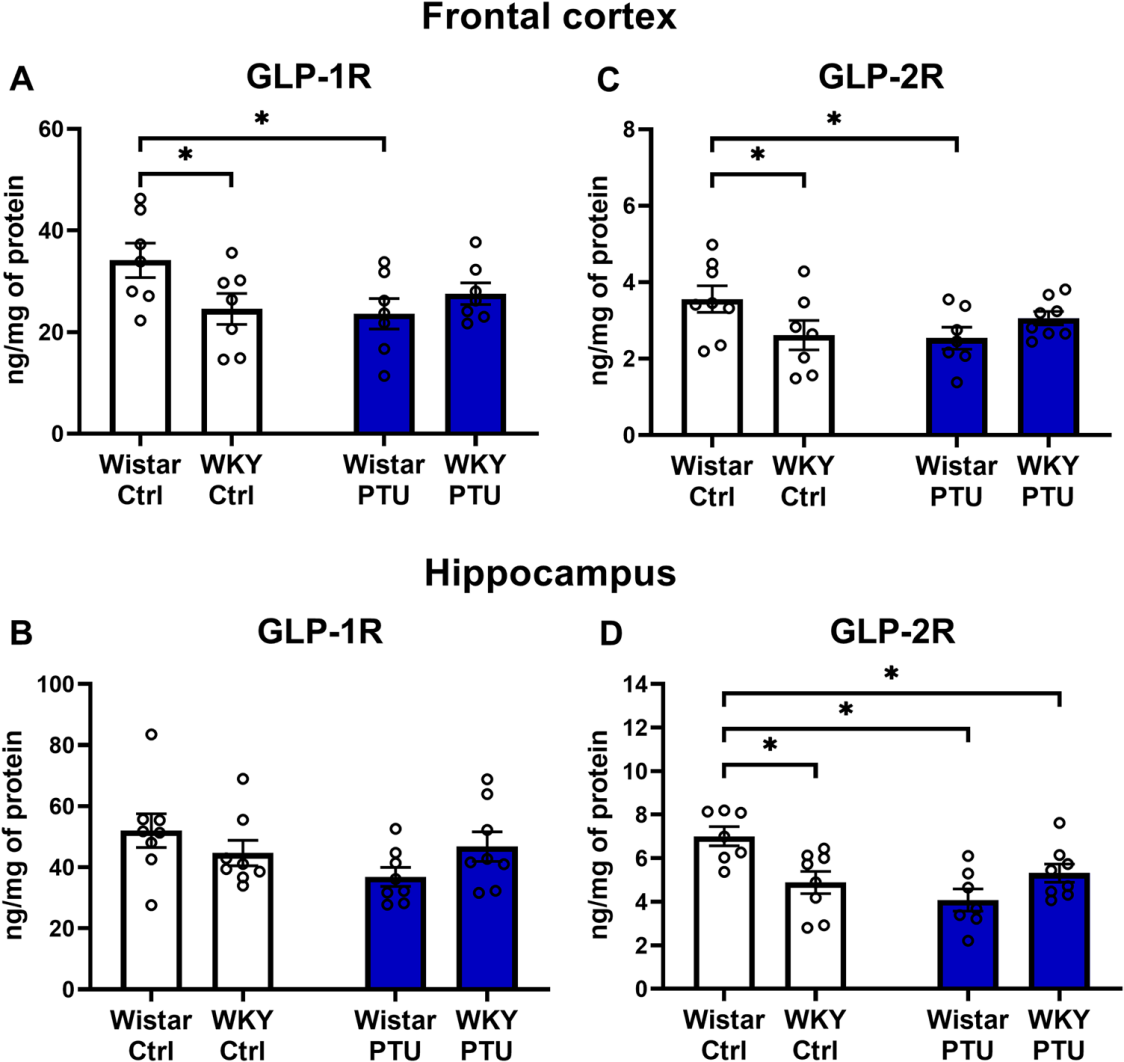
The effects of strain and PTU treatment on the protein levels of GLP-1R **A**, **B** and GLP-2R **C**, **D** in the frontal cortex and hippocampus. The results are expressed as the ng/mg of protein ± SEM. *p < 0.05 vs. the control group (Wistar rats). *n* = 6–8. Statistics: two-way ANOVA followed by Duncan’s test

In the frontal cortex, the concentrations of GLP-1R and GLP-2R were reduced in WKY rats in comparison to the control Wistar rats, and the same effect was induced by the PTU in the Wistar strain (strain × PTU interaction: *F*_1,24_ = 5.33, *p* = 0.030) (Fig. 6A, C). In the hippocampus, such an effect was not observed in GLP-1R, although the GLP-2R level in the hippocampus in WKY rats (both PTU-treated and non-treated) and in the Wistar + PTU group was reduced in comparison to the control (strain × PTU interaction: *F*_1,26_ = 12.66; *p* = 0.001, PTU effect: *F*_1,26_ = 7.04, *p* = 0.013) (Fig. 6B, D).

## Discussion

The present study showed that changes in the markers of cholesterol and/or lipid biosynthesis in a model of depression, hypothyroidism, or the coexistence of these two diseases, mainly indicate a structure-dependent and model-dependent reduction in cholesterol biosynthesis and its uptake.

The models of depression and hypothyroidism used in this study were verified in our previous studies. In addition to the fact that the WKY rats are a well-described model of depression [22], in our research, conducted at the same time as the current study, we also confirmed the presence of pro-depressive behavior in the forced swim test, pro-anxiety behavior in the elevated plus-maze test and memory disturbances in the novel object recognition and novel object location test [14, 31]. Moreover, in WKY rats, we also observed a reduction in long-term potentiation in the dentate gyrus and CA1 region of the hippocampus and in short-term synaptic plasticity in the CA1 region of the hippocampus in electrophysiological studies, which also indicates the presence of memory disturbances in WKY rats [14]. Importantly, in WKY rats, despite the lack of changes in the level of thyroid hormones in the blood, in the frontal cortex, we showed a decrease in the level of T3, the main thyroid hormones receptor (TR*α*), and the level of deiodinase 2, which confirms the theory that in depression the activity of thyroid hormones is weakened in the brain. Also, the hypothyroidism model used in the present study is well described in the literature and verified by us previously by showing a strong reduction in fT3 and fT4 levels and an increase in blood TSH levels in both control animals (Wistar) and the WKY strain [20]. In the present study, we focused on the definition of lipid metabolism in the brain in these models.

The obtained results indicate that the reduction of cholesterol biosynthesis in hypothyroidism occurs especially in the frontal cortex. First of all, lower levels of the mature form of SREBP-2, a transcription factor preferentially involved in cholesterol synthesis, was found in the frontal cortex of hypothyroid rats. In this brain structure, a decreased level of the SREBP-2 precursor was observed in all of the study groups, although only in the Wistar rats receiving PTU was an active form of SREBP-2 that binds to the SRE sequence and activates gene transcription significantly reduced. It was also established that the lack of changes in the level of the mature form of SREBP-2, despite the reduction of its precursor in WKY rats, was not due to changes in the levels of INSIG-1 or INSIG-2, meaning proteins that retain SREBPs in the endoplasmic reticulum and thus prevent their translocation to Golgi apparatus and activation by proteolytic cleavage. The lowering of cholesterol synthesis in the frontal cortex in the Wistar rats with hypothyroidism is also evidenced by decreasing the level of 3-hydroxy 3-methylglutaryl coenzyme A reductase (HMGCR), the key and rate-limiting enzyme of the cholesterol biosynthetic pathway. While changes indicative of lowering cholesterol levels in the frontal cortex were observed in the hypothyroidism model, in the depression model the reduction of the mature, transcriptionally active form of SREBP-2 occurred in the hippocampus. The different regulation of cholesterol homeostasis in individual regions of the brain has previously been described and is mainly associated with regional myelin content, cholesterol turnover and the modulation of synaptic plasticity [25, 26]. In the adult brain, cholesterol is mainly produced in astrocytes and then delivered to neurons from them. The reduction in cholesterol synthesis observed in the current studies was not, however, due to a reduction in the number of astrocytes, as no reduction in GFAP expression, a specific astrocyte marker, was observed in any of the models used. Although most of the cholesterol in the brain is found in the myelin sheaths in oligodendrocytes and in the membranes of nerve cells, it is not only a structural component but also an important factor regulating cell function, including synaptic transmission, synaptogenesis, and neurotransmitter release [20, 27]. In previous studies using the same model of depression as now, we demonstrated a reduction of long-term potentiation (LTP) in the dentate gyrus (DG) and CA1 region of the hippocampus, as well as a reduction in short-term synaptic plasticity [14]. Moreover, it was previously shown in both our behavioral and electrophysiological studies in this model of depression that a decrease in memory processes were linked with a lowered level of acetylcholine in the hippocampus, although using the current results as a basis it seems that these behavioral and synaptic changes can also be due to lowered cholesterol levels.

In the case of SREBP-1, there was a reduction in the level of the precursor form of this transcription factor in the frontal cortex in the hypothyroidism model, and in the hippocampus in the depression-hypothyroidism coexistence model, although these changes did not lead to a reduction in the mature, active form of this factor. These results suggest that in depression and hypothyroidism, changes in lipid metabolism mainly affect cholesterol biosynthesis. A similar relationship as in the case of the levels of SREBPs was also observed in the changes of factors regulated by SREBP-1 and SREBP-2, respectively. In the frontal cortex in the hypothyroidism model, the decrease in the level of the active form of SREBP-2 was accompanied by a reduction in the concentration of factors involved in cholesterol biosynthesis and regulated by this transcription factor, namely 3-hydroxy-3-methylglutaryl-coenzyme A synthase-1 (HMGCS1) and 3-hydroxy-3-methyl-glutaryl-coenzyme A reductase (HMGCR). In the case of the hippocampus, the decrease in the active form of SREBP-2 in WKY rats was accompanied only by a downward trend in HMGCS levels, but this was not a significant change. In turn, in line with the lack of changes in the level of the active form of SREBP-1, there were also no changes in the concentration of stearoyl-CoA desaturase (SCD), an enzyme regulated by this transcription factor, which also confirms that in depression and/or hypothyroidism, biosynthesis of cholesterol rather than the fatty acids is disturbed.

The action of cholesterol depends not only on its synthesis, but also on its uptake through the low density lipoprotein receptor (LDL-R) [28]. The approximately 160 kDa mature form of LDL-R is produced by N- and O-glycosylation of the approximately 120 kDa precursor. Current studies have shown that in a model of coexistence of depression and hypothyroidism, the level of the mature, active form of LDL-R and its precursor are significantly lower in the frontal cortex. A downward trend in the precursor and mature LDL-R forms were also observed in the frontal cortex in the depression model, but a significant decrease in the active form occurred only in the presence of depression and hypothyroidism. Lowering LDL-R concentration may be important in cholesterol regulation of brain function, including induction or exacerbation changes important in the pathogenesis of CNS diseases. For example, it has been found that cholesterol uptake through this receptor may be a mechanism of *β*-amyloid peptide elimination from the brain [28]. Some data indicate the role of this receptor in the pathogenesis of depression, since LDL receptor deleted mice show a depressive-like behavior possibly due to hyperactivity of brain monoamine oxidase A and B, i.e. monoamine metabolizing enzymes [29]. LDL-R knockout mice display an increased total cholesterol plasma level, hence the same change as seen in humans with familial hypercholesterolemia caused by a genetic defect in the gene encoding the LDL receptor [30]. In previous studies, we observed an increase in plasma total cholesterol in the Wistar and WKY rats with hypothyroidism and the LDL-cholesterol fraction in the depression and hypothyroidism model, and the highest level in the model of coexistence of these two diseases [31], which correlates with the reduction of LDL-R currently demonstrated. The differences in LDL-R levels, along with disturbances in the biosynthesis of cholesterol in the brain, may to some extent explain the inconsistent clinical data so far regarding the relationship between depression and peripheral cholesterol levels. There are data showing a relationship between low cholesterol and depression, as well as a relationship between depression and hypercholesterolemia [27, 32], therefore it is possible that the level of LDL-R in the brain, and not only peripheral or central cholesterol concentrations, may be a significant change predisposing to the development of depression. Importantly, in the model of depression caused by LDL-R knockout in mice, the authors suggested that increased MAO activity may be the cause of the intensification of oxidative stress and mitochondrial dysfunction in brain cells. As in the LDL-R knockout-induced depression model, changes indicative of damage to brain cells or disturbed neuronal plasticity have often been observed in other animal models of depression [33, 34]. In the WKY rats, used in the present study as a model of depression, we showed a reduction in the level of synaptic protein SNAP-25 in both the frontal cortex and the hippocampus, and in the case of the hippocampus, the reduction was greater in the model of comorbidity of depression and hypothyroidism. SNAP-25, a synaptosomal-associated protein, participates in the regulation of synaptic vesicle exocytosis, modulates voltage-gated calcium channels and controls the secretion of neurotransmitters [35]. Thus, the reduction in the level of SNAP-25 in the hippocampus observed in the model of coexistence of depression and hypothyroidism may be an essential cause of the weakening of short-term synaptic plasticity, which we demonstrated previously with electrophysiological methods in this model, and which depends on the presynaptic mechanisms of neurotransmitter release [14]. In the hippocampus, in addition to a decrease in the level of SNAP-25 in the model of depression and the coexistence of depression and hypothyroidism, there was also a decrease in the level of the receptor for glucagon-like peptide 2 (GLP-2R) in all three tested models. Since this peptide, in addition to regulating food intake, has anxiolytic, neuroprotective and memory-enhancing effects [36, 37], downregulation of its receptor might thus be one of the causes of anxiety-like behaviors and disruptions in spatial memory described previously in WKY animals and in the model of coexistence of depression and hypothyroidism [14]. Moreover, as with GLP-1, who’s neuroprotective, improving memory and cognitive functions as well as enhancing neurogenesis and synaptic function effects are well described [37], similar effects of GLP-2 have also been demonstrated. For example, it has been found, that increase in GLP-2R expression enhances neurogenesis in the dentate gyrus, neuronal activity, and the density of dendritic spines in the hippocampal neurons [38].

Changes indicating a disturbance of synaptic plasticity and/or induction of nerve cell damage, such as decreased levels of SNAP-25 protein and GLP-1R and GLP-2R receptors, were also present in the frontal cortex, hence its being a structure in which disturbances like those found in the hippocampus are often observed in depression.

Summarizing the changes shown in the tested models, it can be said that in the depression model, changes such as lowering the level of the active form of SREBP-2, SNAP-25 and GLP-2R in the hippocampus and SNAP-25, GLP-1R and GLP-2R in the frontal cortex indicate disturbances in synaptic plasticity in both these brain regions as well as lowering molecules involved in cholesterol synthesis in the hippocampus. Potential cholesterol reduction in the brain, in addition to adversely affecting synaptic plasticity and neurotransmitter release, may also lead to limitations in the synthesis of sex hormones and neurosteroids, including compounds such as allopregnanolone, progesterone or dehydroepiandrosterone, which exhibit antidepressant activity [39]. A marked reduction in cholesterol biosynthesis was also observed in hypothyroidism by a decrease in the level of SREBP-2 and its regulated enzymes in the frontal cortex, whereas changes in the expression of SNAP-25 and GLP-1 and 2 receptors indicate a weakening of neuroprotective mechanisms. In the model of the coexistence of depression and hypothyroidism, changes in markers of synaptic plasticity/neuroprotection were similar to those observed in the models of each of these diseases individually. However, hypothyroid-induced reductions in cholesterol synthesis markers in the frontal cortex as well as a decrease in SREBP-2 in the hippocampus in depression model were weaker in the model of the coexistence of depression and hypothyroidism. This effect is difficult to explain because the interaction between thyroid hormones and cholesterol synthesis is complex. SREBP-2 is a key factor not only in cholesterol synthesis, but also in the synthesis of thyroid hormones and, on the other hand, this transcription factor is regulated by thyroid hormones [40]. A significant change occurring in the frontal cortex only in the model of the coexistence of depression and hypothyroidism was a decrease in LDL-R concentration and, consequently, cellular cholesterol uptake. This may be an important alteration because, in light of the current research, lowering the level of LDL-R in the brain is associated with anxiety- and depression-like behaviors.

## Supplementary Information

Below is the link to the electronic supplementary material.

Supplementary file1 (PDF 551 KB)

## Data Availability

The datasets generated during the current study are available from the corresponding author on reasonable request from qualified researchers.

## Abbreviations

CNS: Central nervous system
GFAP: Glial fibrillary acidic protein
GLP-1R: Glucagon-like peptide-1 receptor
GLP-2R: Glucagon-like peptide-2 receptor
HMGCR: 3-Hydroxy-3-methylglutaryl coenzyme A reductase
HMGCS: 3-Hydroxy-3-methylglutaryl coenzyme A synthase
HPA: Hypothalamic–pituitary–adrenal axis
HRP: Horseradish peroxidase
INSIG-1: Insulin-induced gene 1 protein
INSIG-2: Insulin-induced gene 2 protein
LDL: Low-density lipoprotein
LDL-R: Low-density lipoprotein receptor
PTU: Propylthiouracil
SCAP: SREBP cleavage activating protein
SCD: Stearoyl coenzyme A desaturase
SDS: Sodium dodecyl sulfate
SNAP-25: Synaptosomal associated protein 25 kDa
SRE: Sterol regulatory element
SREBP-1: Sterol regulatory element-binding protein 1
SREBP-2: Sterol regulatory element-binding protein 2
TBS: Tris-buffered saline
WKY: Wistar-Kyoto
