# Supplementary material for: Changes in regulators of lipid metabolism in the brain: a study of animal models of depression and hypothyroidism
Source: Pharmacol Rep. 2022 Aug 11;74(5):859–70. doi: 10.1007/s43440-022-00395-8 (PMC9584974; doi:10.1007/s43440-022-00395-8)
Supplement: Supplementary file 1 — Supplementary file1 (PDF 551 KB) [file 43440_2022_395_MOESM1_ESM.pdf]

Frontal cortex
GFAP membrane 1 and membrane 2

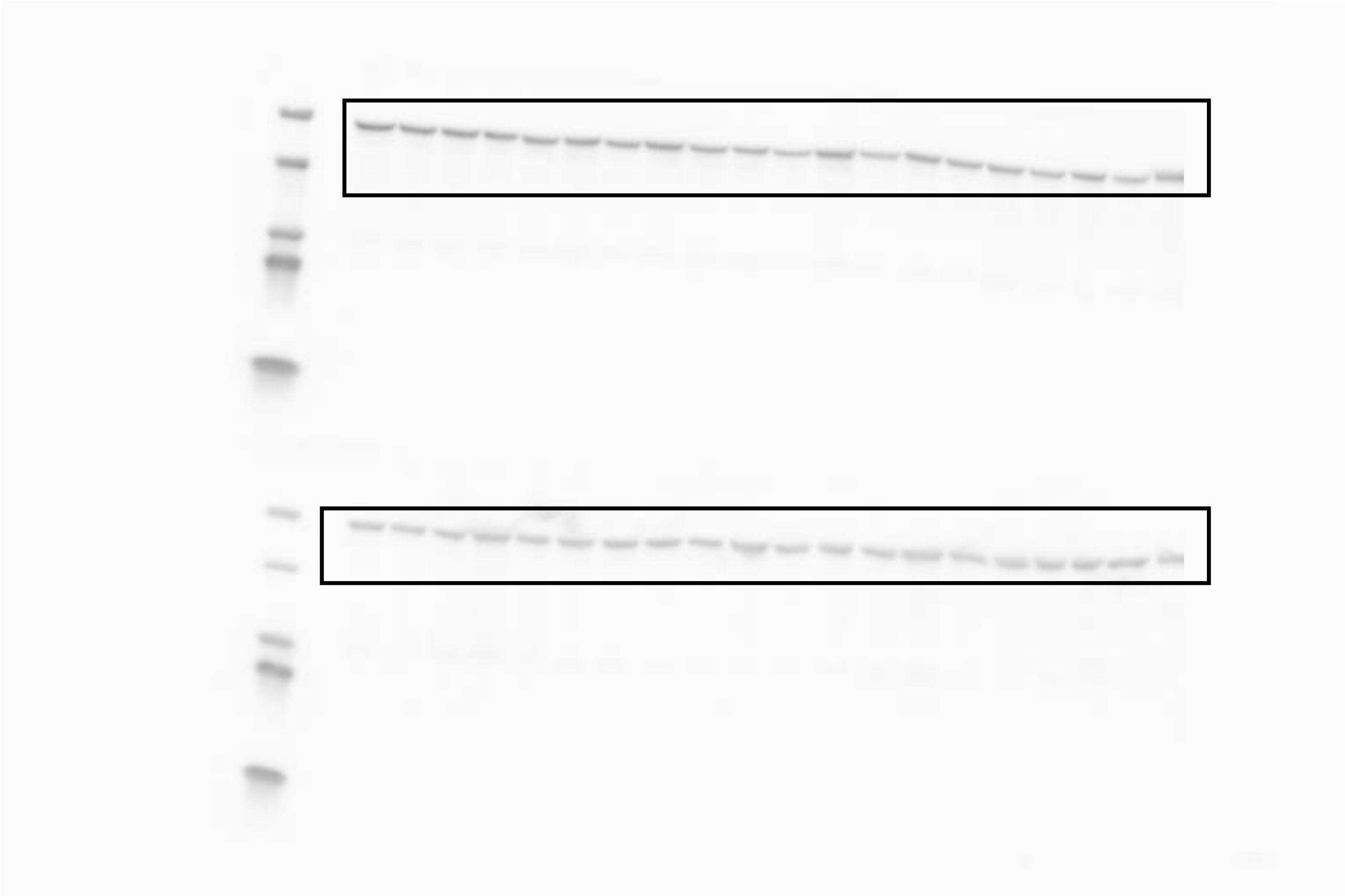

Frontal cortex
Vinculin to GFAP membrane 1 and membrane 2

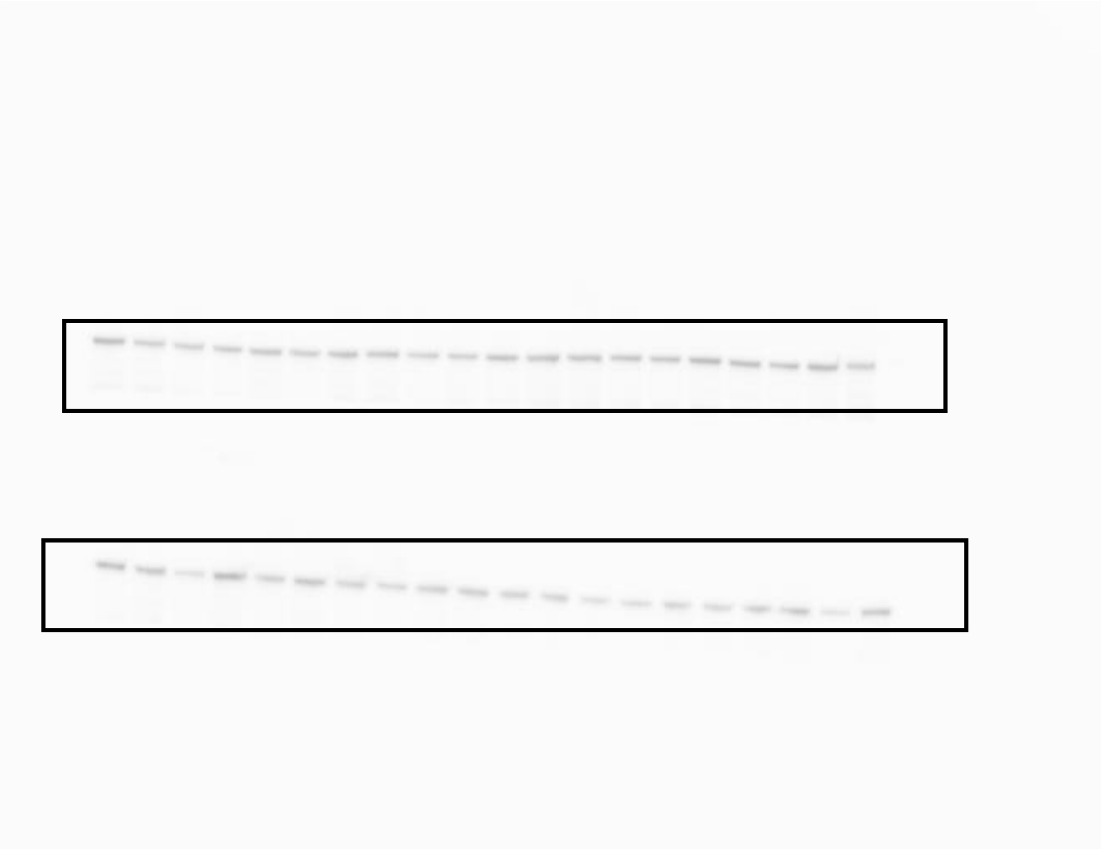

The bands from the left: Wistar, WKY, Wistar PTU, WKY PTU, Wistar, WKY, Wistar PTU, WKY PTU, Wistar, WKY, Wistar PTU, WKY PTU, Wistar, WKY, Wistar PTU, WKY PTU, Wistar, WKY, Wistar PTU, WKY PTU

Hippocampus
GFAP membrane 1 and membrane 2

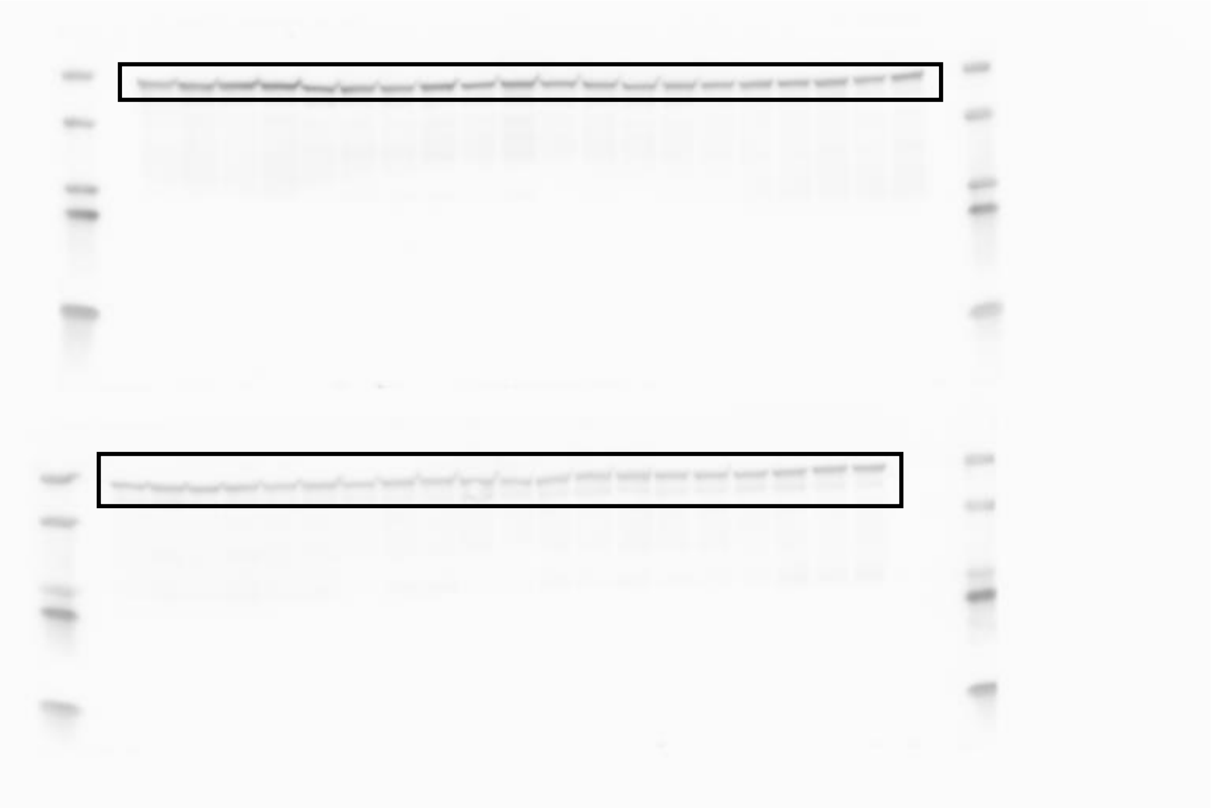

Hippocampus
Vinculin to GFAP membrane 1 and membrane 2

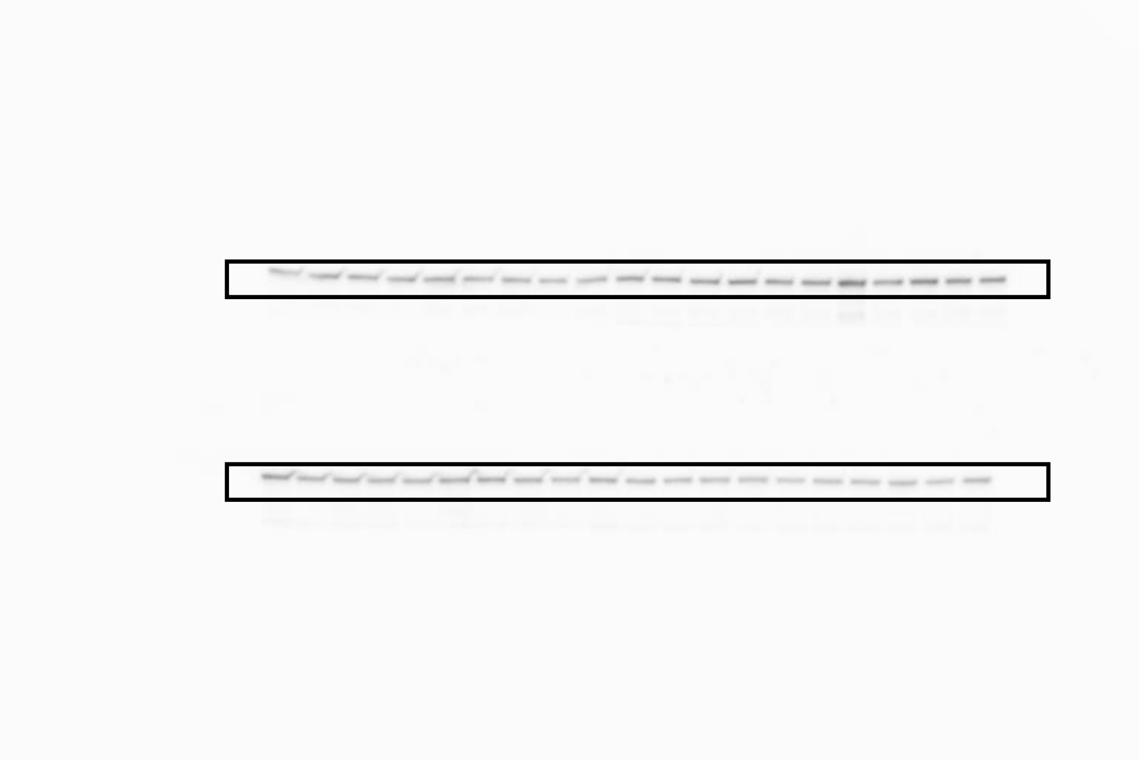

The bands from the left: Wistar, WKY, Wistar PTU, WKY PTU, Wistar, WKY, Wistar PTU, WKY PTU, Wistar, WKY, Wistar PTU, WKY PTU, Wistar, WKY, Wistar PTU, WKY PTU, Wistar, WKY, Wistar PTU, WKY PTU

Frontal cortex
SREBP 1 membrane 1

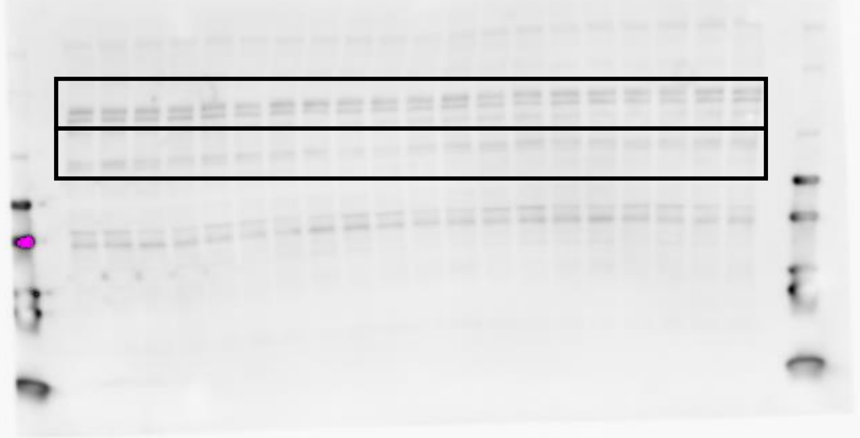

precursor
mature

Frontal cortex
SREBP 1 membrane 2

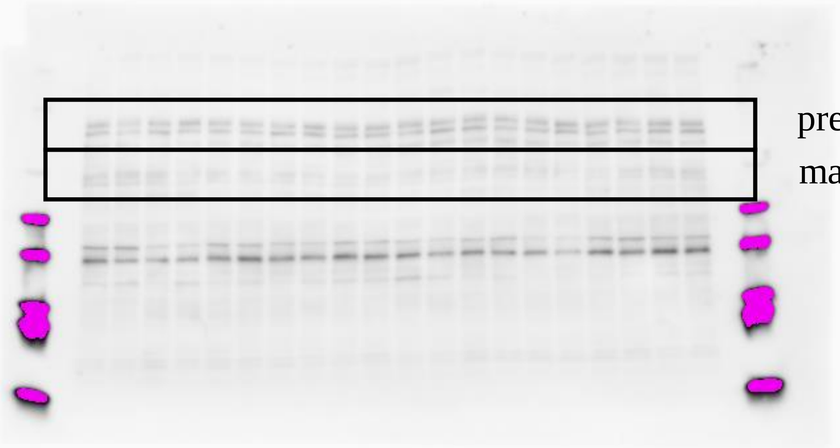

precursor
mature

Frontal cortex
Vinculin to SREBP1 membrane 1 and membrane 2

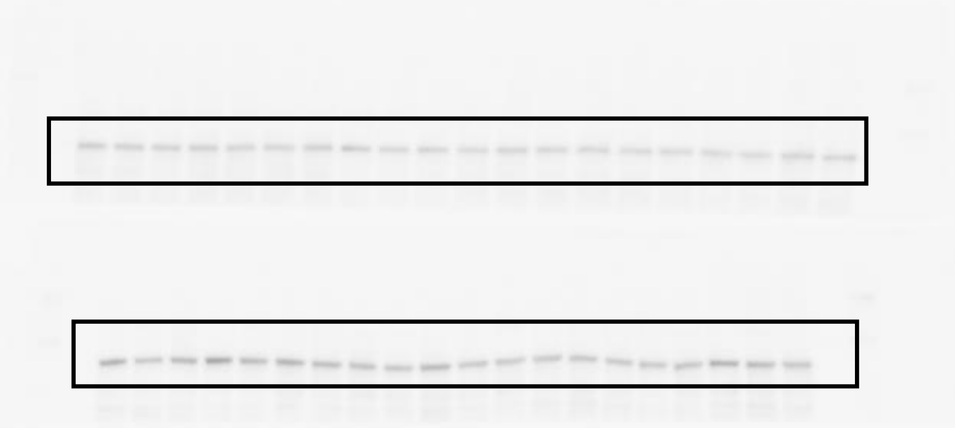

The bands from the left: Wistar, Wistar PTU, WKY, WKY PTU, Wistar, Wistar PTU, WKY, WKY PTU, Wistar, Wistar PTU, WKY, WKY PTU, Wistar, Wistar PTU, WKY, WKY PTU, Wistar, Wistar PTU, WKY, WKY PTU

Hippocampus
SREBP 1 membrane 1

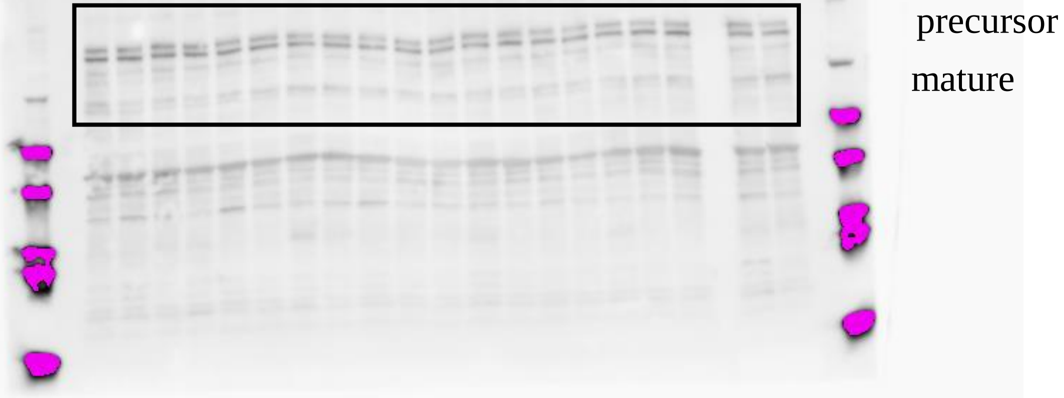

Hippocampus
SREBP 1 membrane 2

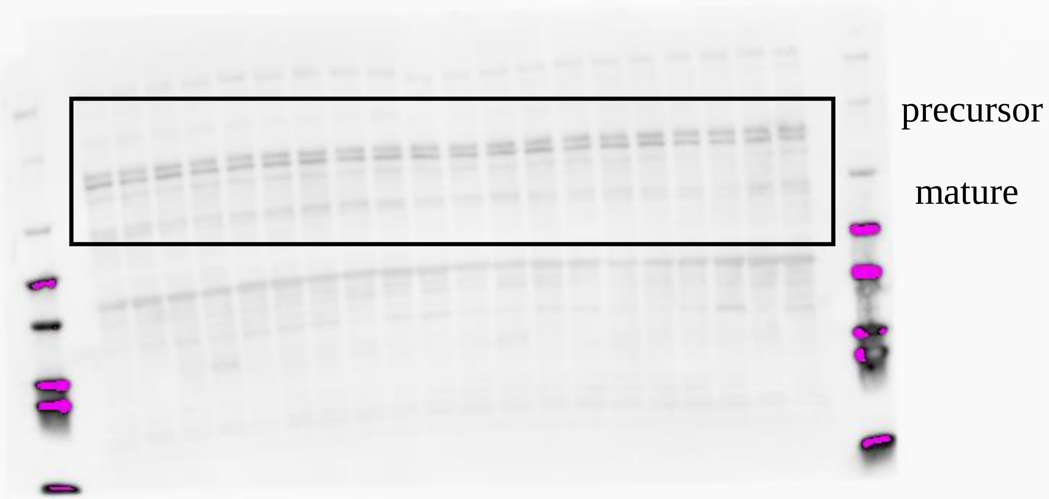

Hippocampus
Vinculin to SREBP1 membrane 1 and membrane 2

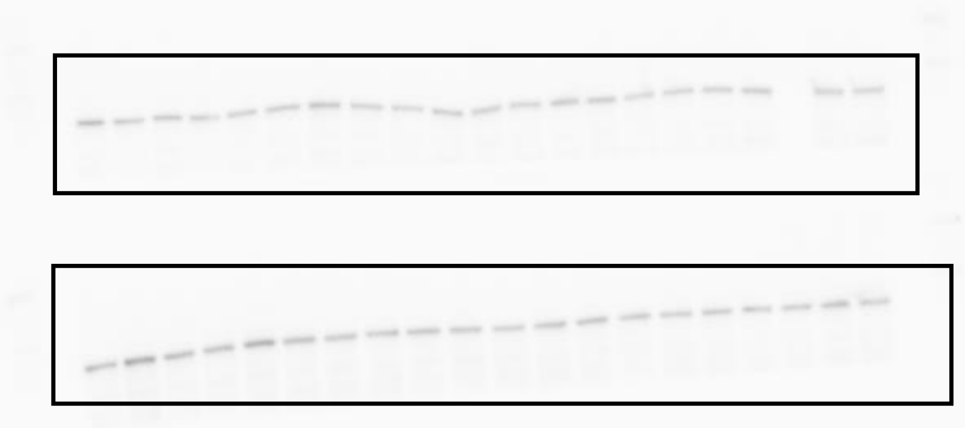

The bands from the left: Wistar, Wistar PTU, WKY, WKY PTU, Wistar, Wistar PTU, WKY, WKY PTU, Wistar, Wistar PTU, WKY, WKY PTU, Wistar, Wistar PTU, WKY, WKY PTU, Wistar, Wistar PTU, WKY, WKY PTU

Frontal cortex
SREBP 2 membrane 1

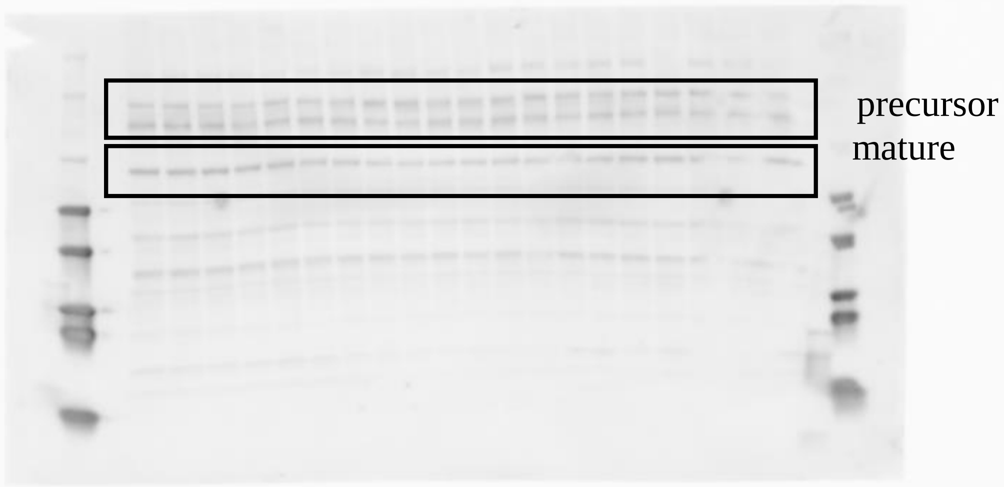

Frontal cortex
SREBP 2 membrane 2

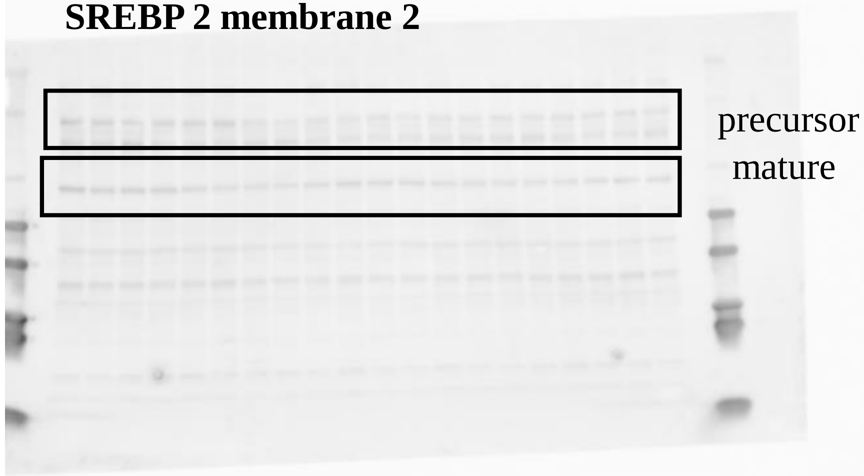

Frontal cortex
Vinculin to SREBP 2 membrane 1 and membrane 2

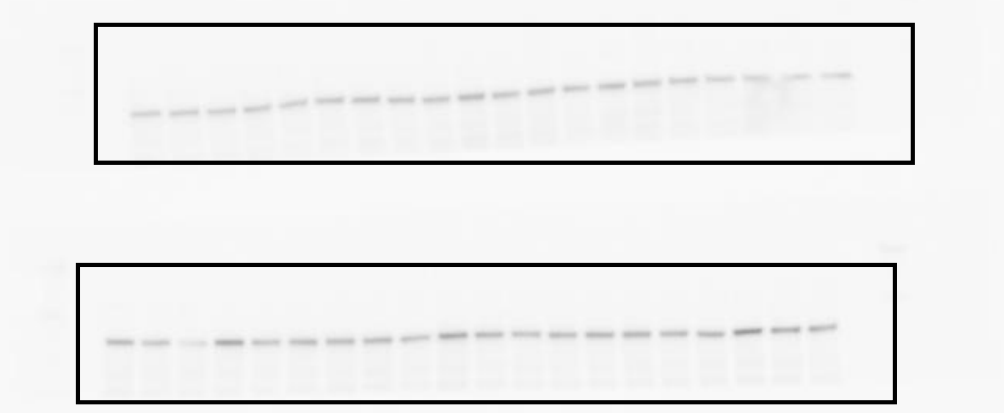

The bands from the left: Wistar, Wistar PTU, WKY, WKY PTU, Wistar, Wistar PTU, WKY, WKY PTU, Wistar, Wistar PTU, WKY, WKY PTU, Wistar, Wistar PTU, WKY, WKY PTU, Wistar, Wistar PTU, WKY, WKY PTU

Hippocampus
SREBP 2 membrane 1

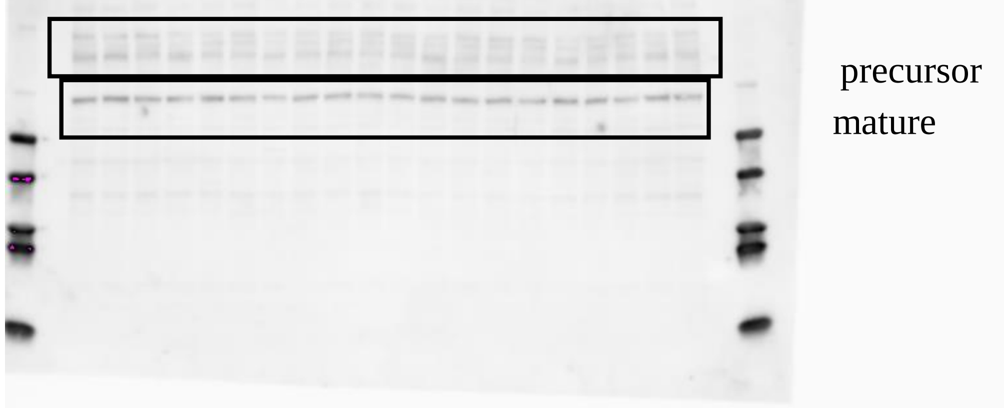

Hippocampus
SREBP 2 membrane 2

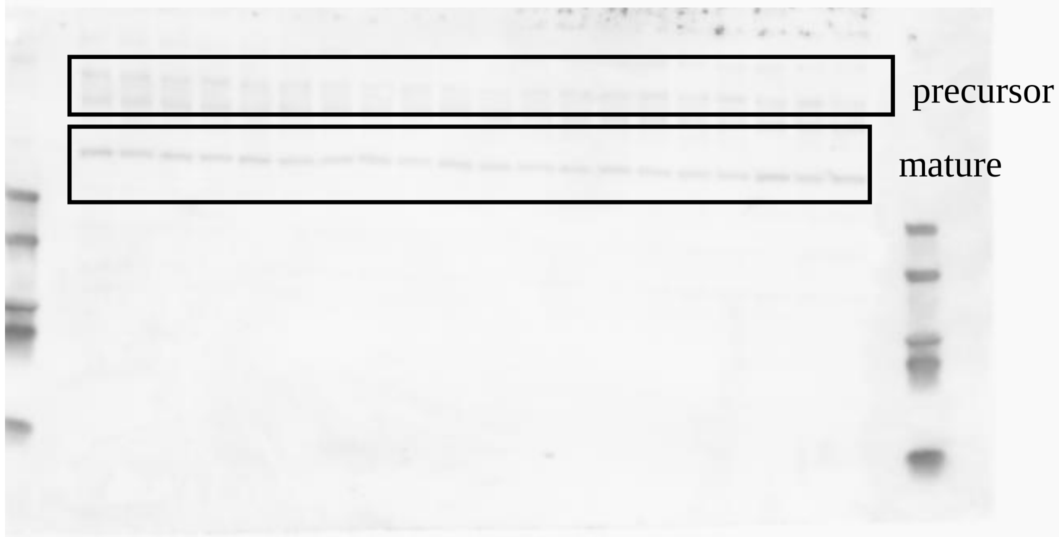

Hippocampus
Vinculin to SREBP 2 membrane 1 and membrane 2

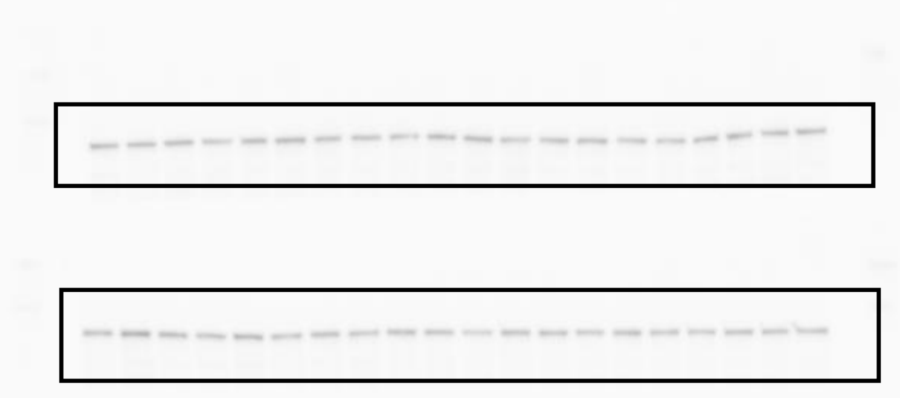

The bands from the left: Wistar, Wistar PTU, WKY, WKY PTU, Wistar, Wistar PTU, WKY, WKY PTU, Wistar, Wistar PTU, WKY, WKY PTU, Wistar, Wistar PTU, WKY, WKY PTU, Wistar, Wistar PTU, WKY, WKY PTU

Frontal cortex
LDL-R membrane 1 and membrane 2

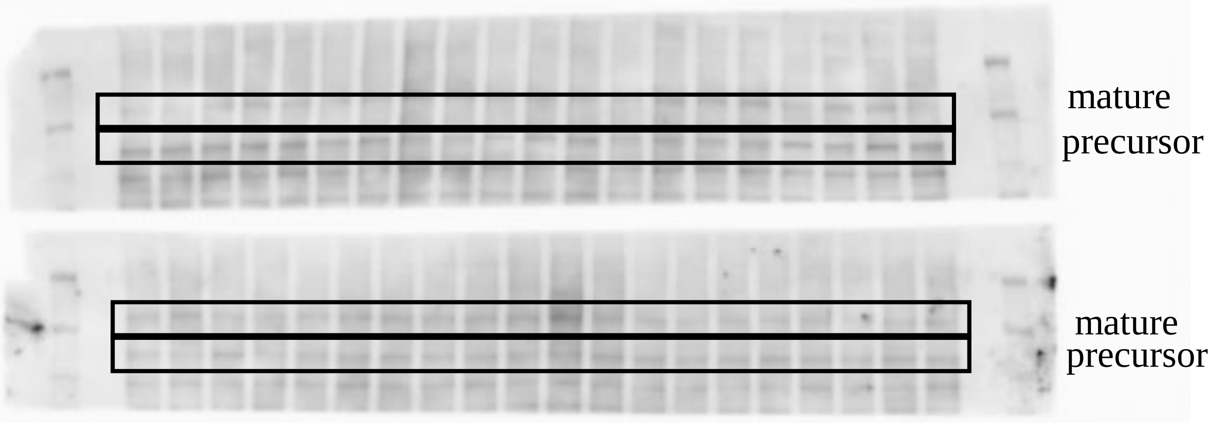

Frontal cortex
B-actin to LDL-R membrane 1

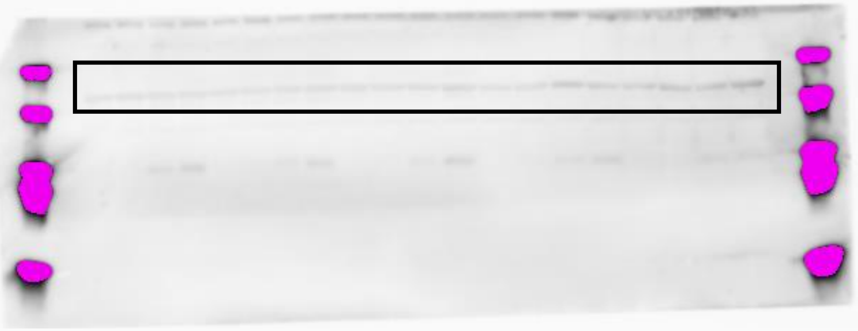

Frontal cortex
B-actin to LDL-R membrane 2

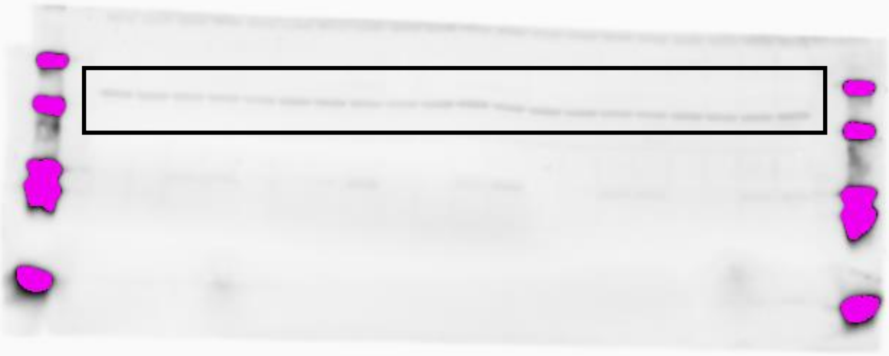

The bands from the left: Wistar, Wistar PTU, WKY, WKY PTU, Wistar, Wistar PTU, WKY, WKY PTU, Wistar, Wistar PTU, WKY, WKY PTU, Wistar, Wistar PTU, WKY, WKY PTU, Wistar, Wistar PTU, WKY, WKY PTU

Hippocampus
LDL-R membrane 1 and membrane 2

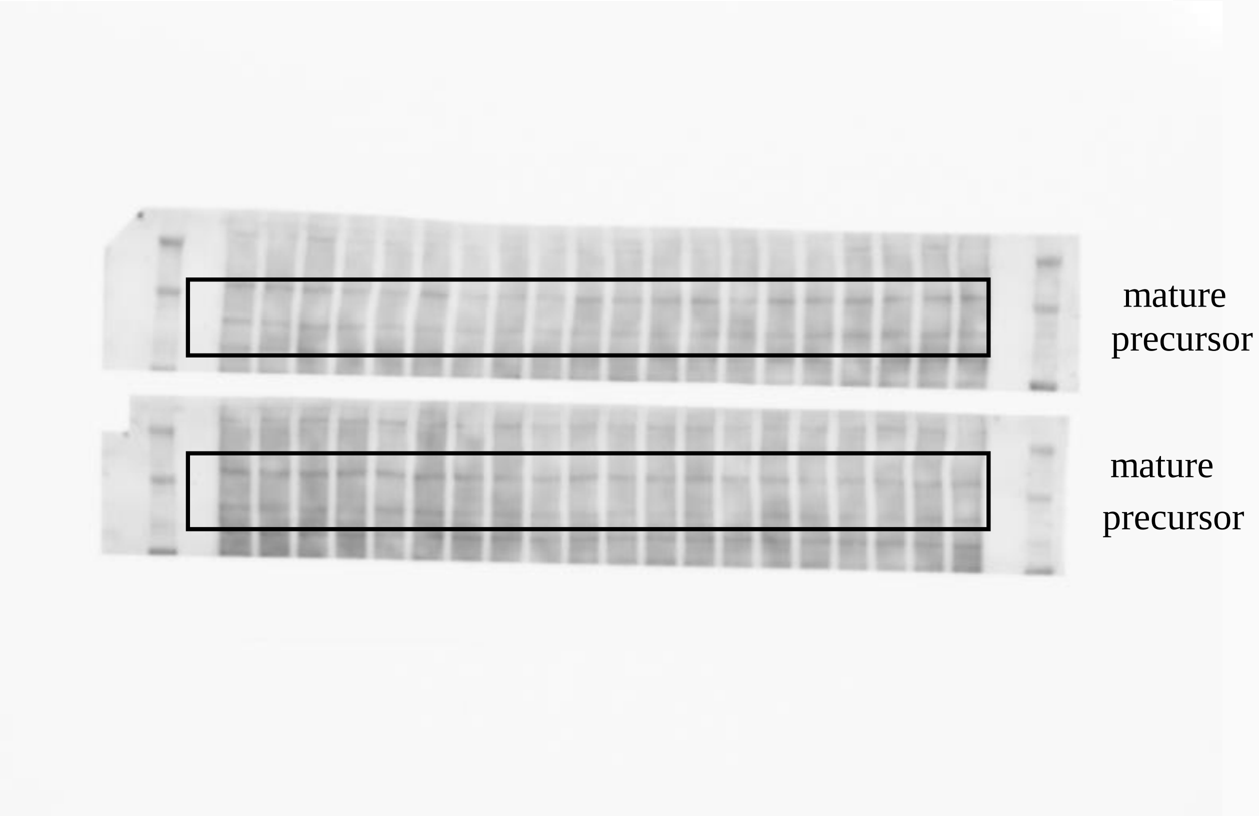

Hippocampus
B-actin to LDL-R membrane 1 and membrane 2

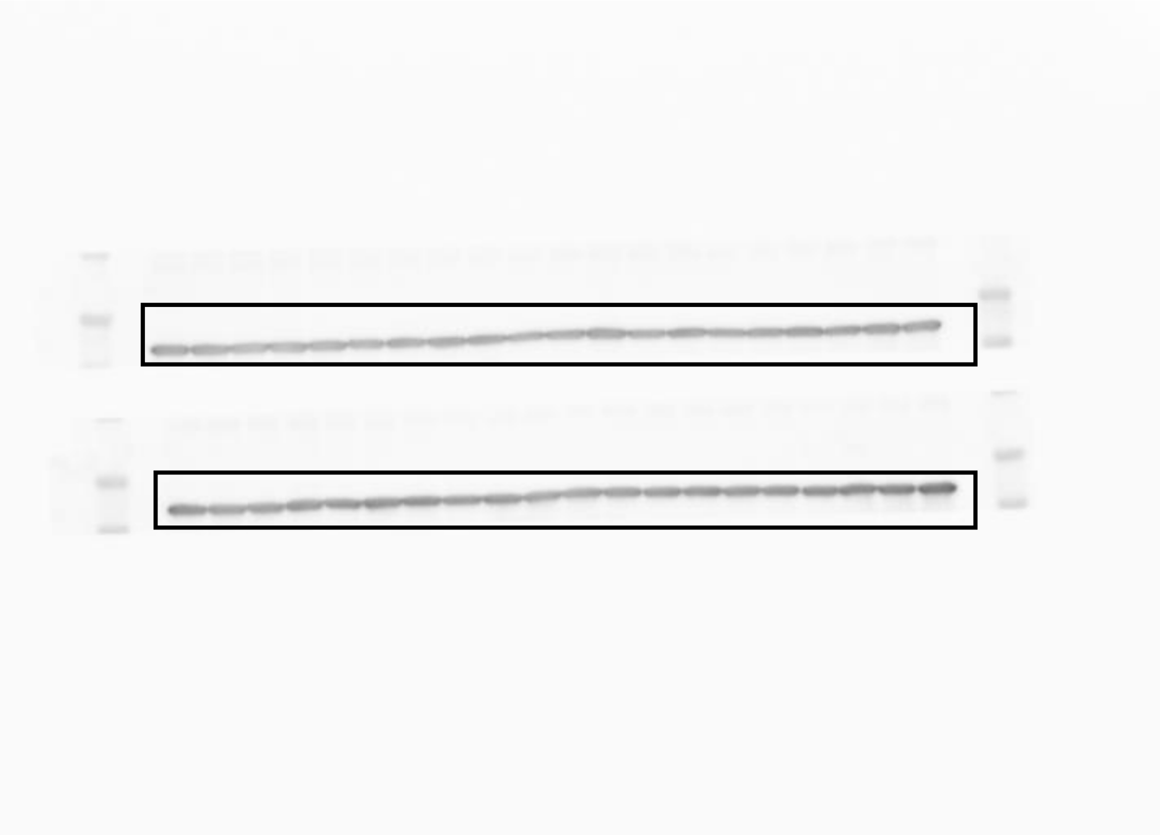

The bands from the left: Wistar, Wistar PTU, WKY, WKY PTU, Wistar, Wistar PTU, WKY, WKY PTU, Wistar, Wistar PTU, WKY, WKY PTU, Wistar, Wistar PTU, WKY, WKY PTU, Wistar, Wistar PTU, WKY, WKY PTU

Frontal cortex
INSIG-1 membrane 1 and membrane 2

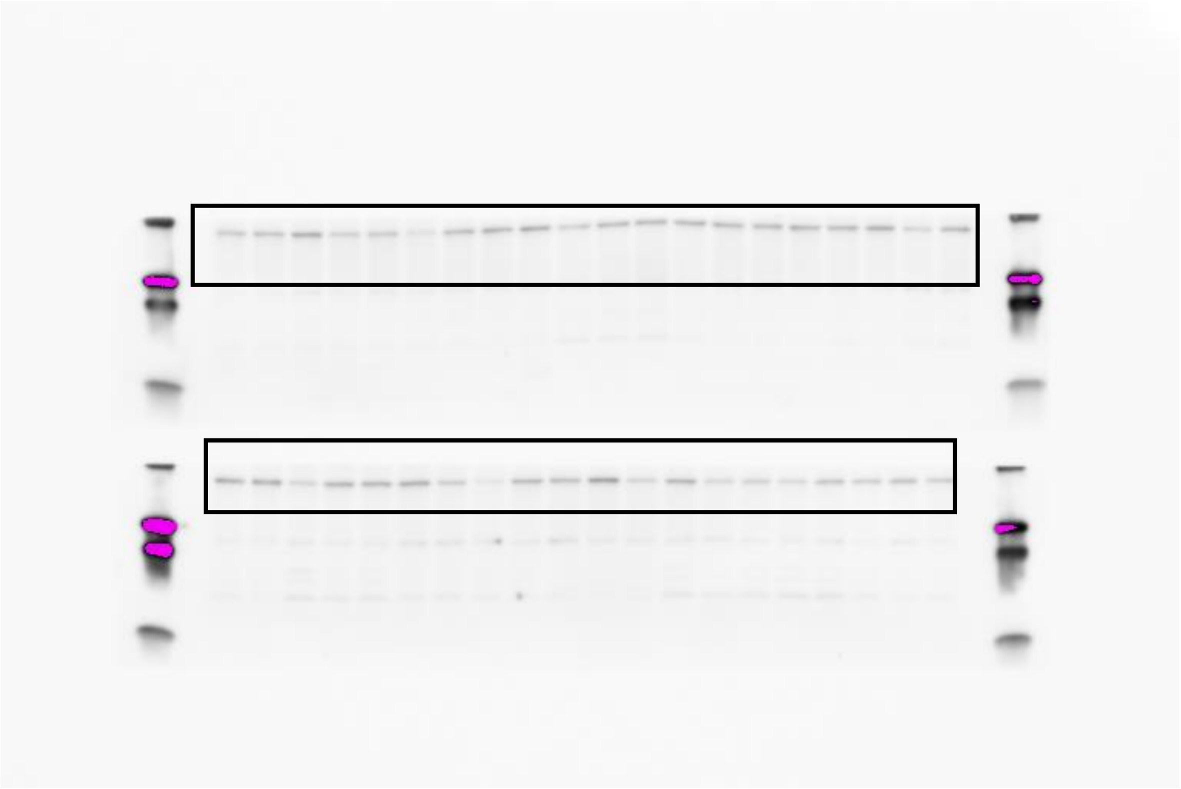

Frontal cortex
B-actin to INSIG-1 membrane 1 and membrane 2

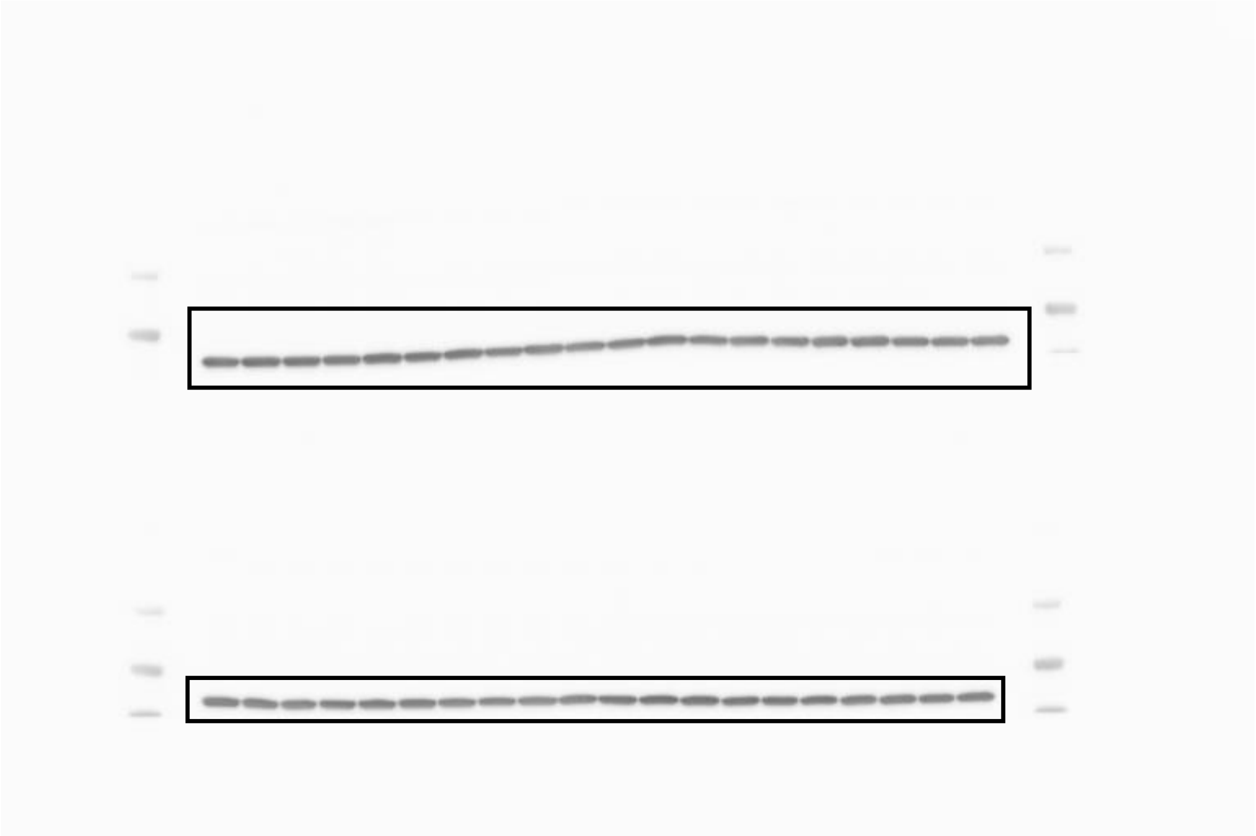

The bands from the left: Wistar, Wistar PTU, WKY, WKY PTU, Wistar, Wistar PTU, WKY, WKY PTU, Wistar, Wistar PTU, WKY, WKY PTU, Wistar, Wistar PTU, WKY, WKY PTU, Wistar, Wistar PTU, WKY, WKY PTU

Hippocampus
INSIG-1 membrane 1 and membrane 2

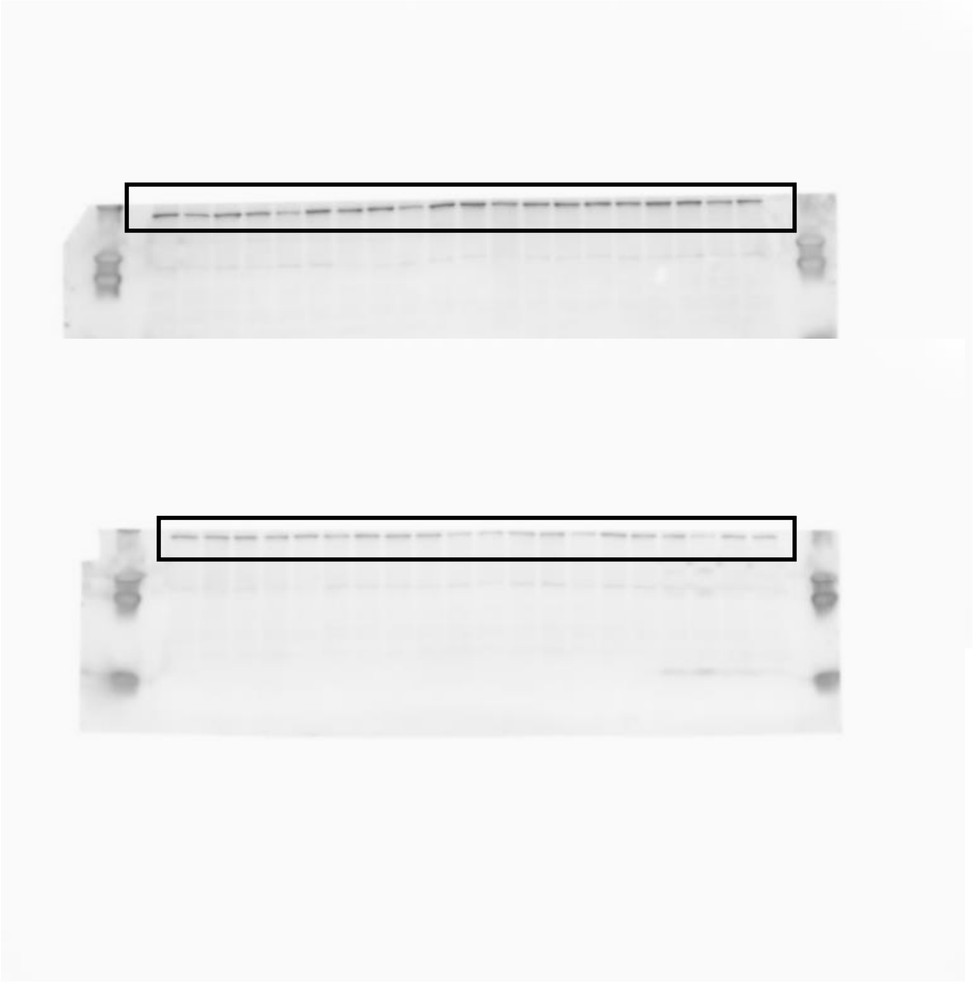

Hippocampus
B-actin to INSIG-1 membrane 1 and membrane 2

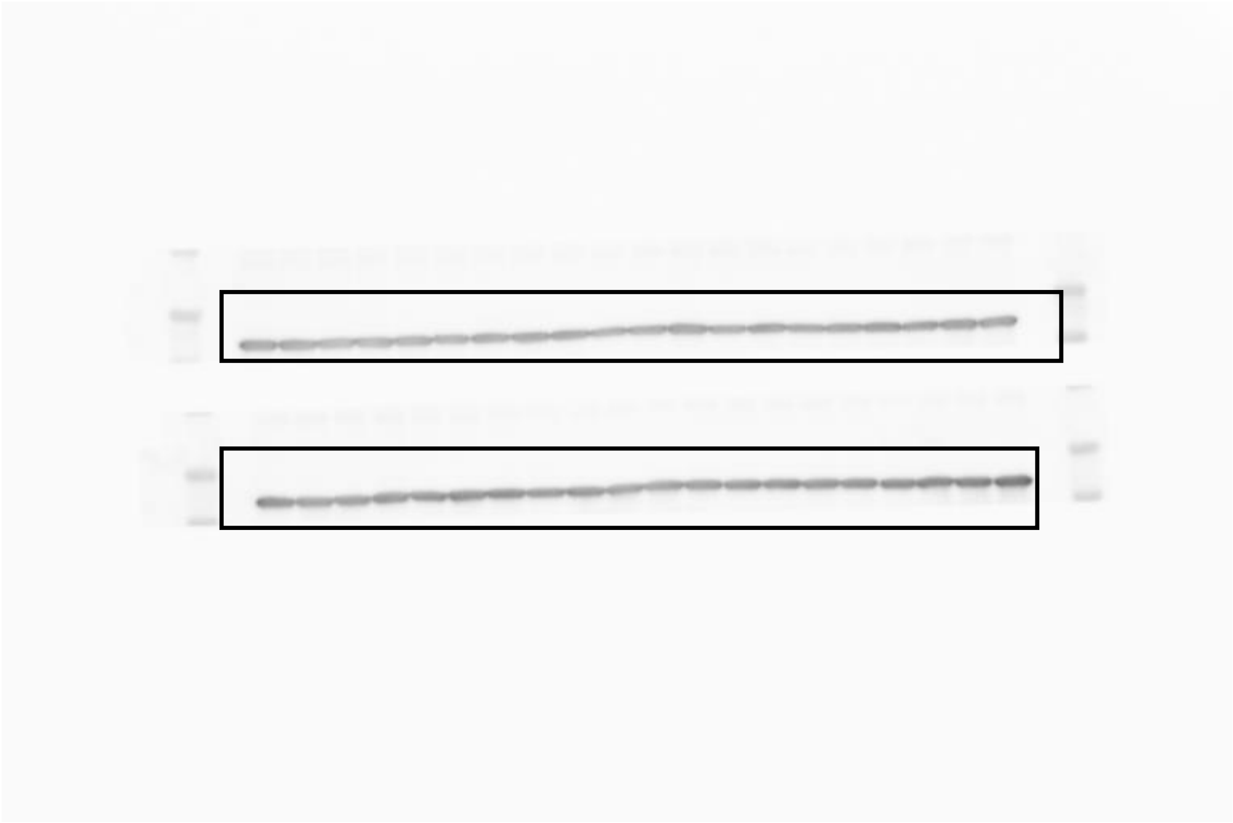

The bands from the left: Wistar, Wistar PTU, WKY, WKY PTU, Wistar, Wistar PTU, WKY, WKY PTU, Wistar, Wistar PTU, WKY, WKY PTU, Wistar, Wistar PTU, WKY, WKY PTU, Wistar, Wistar PTU, WKY, WKY PTU

Frontal cortex
INSIG-2 membrane 1 and membrane 2

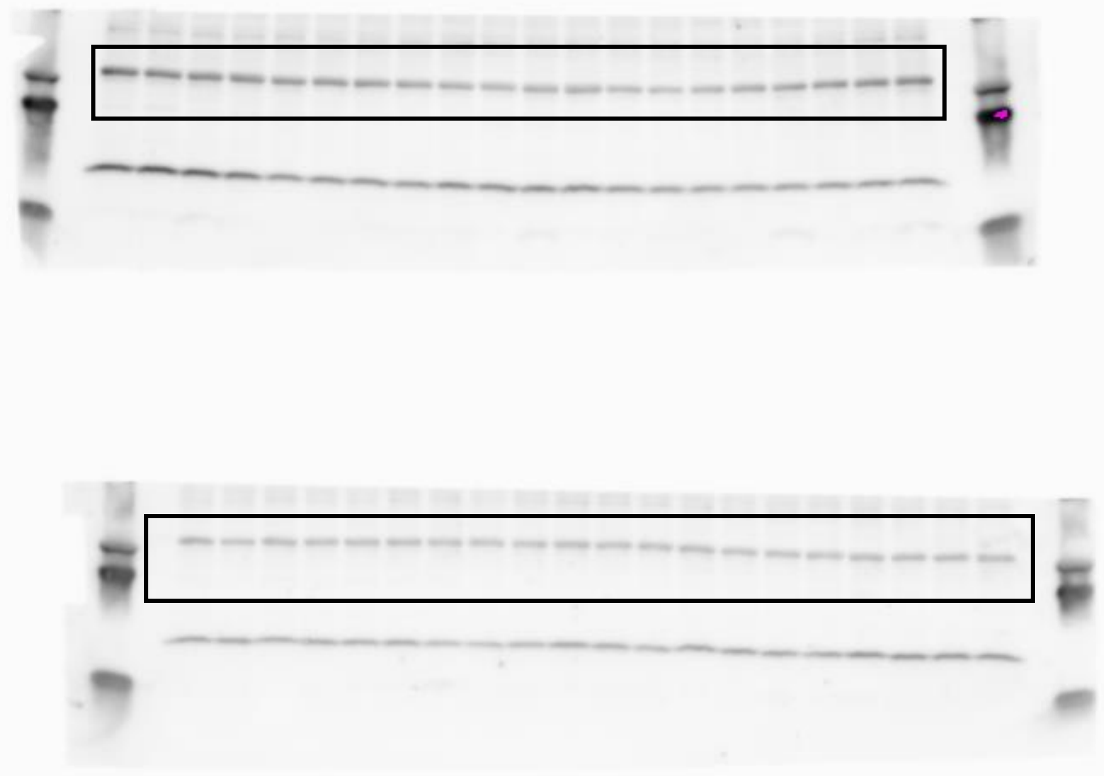

Frontal cortex
B-actin to INSIG-2 membrane 1 and membrane 2

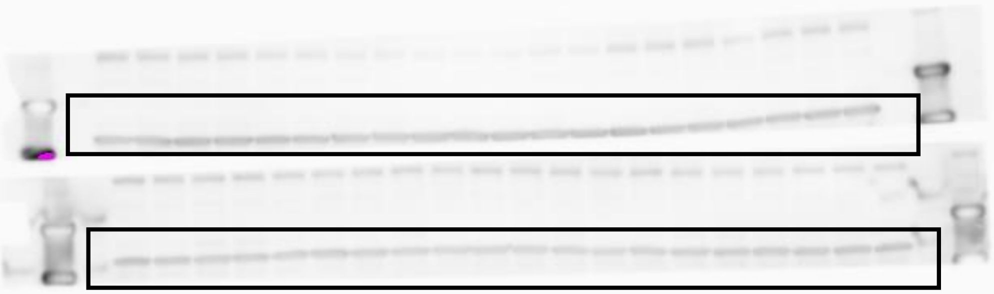

The bands from the left: Wistar, Wistar PTU, WKY, WKY PTU, Wistar, Wistar PTU, WKY, WKY PTU, Wistar, Wistar PTU, WKY, WKY PTU, Wistar, Wistar PTU, WKY, WKY PTU, Wistar, Wistar PTU, WKY, WKY PTU

Hippocampus
INSIG-2 membrane 1 and membrane 2

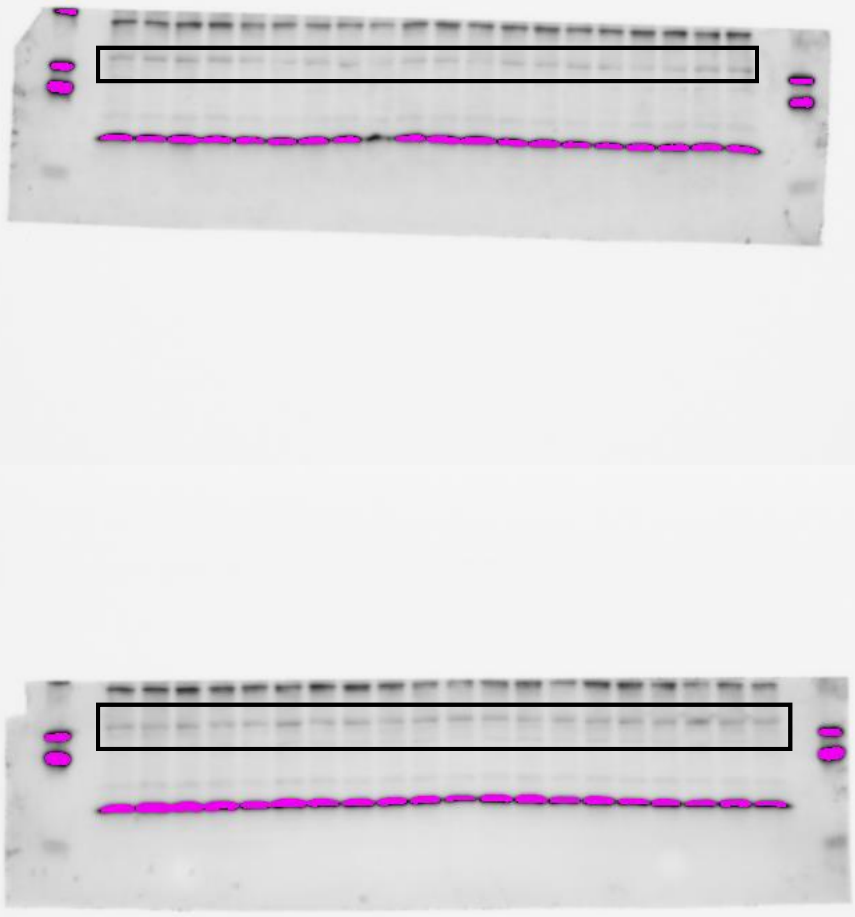

Hippocampus
B-actin to INSIG-2 membrane 1 and membrane 2

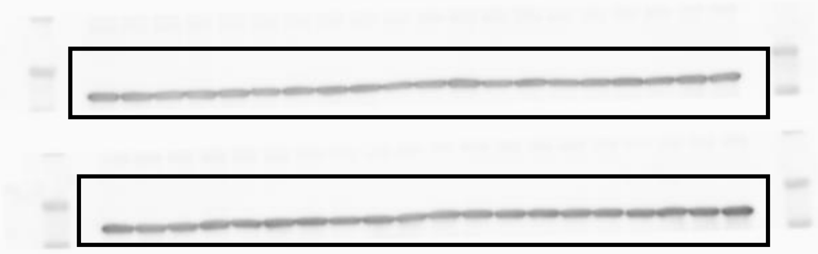

The bands from the left: Wistar, Wistar PTU, WKY, WKY PTU, Wistar, Wistar PTU, WKY, WKY PTU, Wistar, Wistar PTU, WKY, WKY PTU, Wistar, Wistar PTU, WKY, WKY PTU, Wistar, Wistar PTU, WKY, WKY PTU

not all WB samples (presented on pages 1-12) were analyzed (poor quality or outliers were not analyzed)
